# Mechanism of D-type cyclin recognition by the AMBRA1 E3 ligase receptor

**DOI:** 10.1126/sciadv.adu8708

**Published:** 2025-05-23

**Authors:** Yang Wang, Ming Liu, Shan Wang, Xinyi Mai, Xi Wang, Fei Teng, Tianrui Lyu, Ming-Yuan Su, Goran Stjepanovic

**Affiliations:** ^1^ Kobilka Institute of Innovative Drug Discovery, School of Medicine, The Chinese University of Hong Kong, Shenzhen, Guangdong 518172, China.; ^2^ Department of Biochemistry, School of Medicine, Southern University of Science and Technology, Shenzhen, Guangdong 518055, China.; ^3^ Key University Laboratory of Metabolism and Health of Guangdong, Southern University of Science and Technology, Shenzhen, Guangdong 518055, China.; ^4^ Institute for Biological Electron Microscopy, Southern University of Science and Technology, Shenzhen, Guangdong 518055, China.; ^5^ SUSTech Homeostatic Medicine Institute, School of Medicine, Southern University of Science and Technology, Shenzhen, Guangdong 518055, China.

## Abstract

AMBRA1 is a tumor suppressor protein that functions as a substrate receptor in the ubiquitin conjugation system and regulates the stability of D-type cyclins and cell proliferation. Here, we present the cryo-EM structure of cyclin D1–bound AMBRA1-DDB1 complex at 3.55-Å resolution. The structure reveals a substrate interaction surface on the AMBRA1 WD40 domain that specifically binds to the C-terminal region of D-type cyclins. This interaction is dependent on the phosphorylation of Thr^286^ residue in the C-terminal phosphodegron site of D-type cyclins. The phosphodegron motif folds into a turn-like conformation, followed by a 3_10_ helix that promotes its assembly with AMBRA1. In addition, we show that AMBRA1 mutants, which are defective in cyclin D1 binding, lead to cyclin D1 accumulation and DNA damage. Understanding the AMBRA1–D-type cyclin structure enhances the knowledge of the molecular mechanisms that govern the cell cycle control and may lead to potential therapeutic approaches for cancers linked to abnormal cyclin D activity.

## Teaser

Cryo-EM structure reveals how AMBRA1 binds phosphorylated cyclin D1, regulating its stability and controlling cell proliferation.

## INTRODUCTION

The ubiquitin-proteasome system (UPS) is a highly regulated and intricate cellular machinery responsible for maintaining protein homeostasis and controlling key cellular processes. It involves a series of enzymatic reactions that results in the attachment of ubiquitin molecules to target proteins, marking them for degradation by the proteasome. The UPS plays a crucial role in removing aberrant or damaged proteins that could be harmful to the cell. In addition, it regulates the turnover of numerous proteins involved in various cellular functions, such as cell cycle progression, signal transduction, and protein quality control. One specific aspect of cell cycle regulation where the UPS is highly involved is the control of cyclin levels and cyclin-dependent kinases (CDKs) activity (*1*). Cyclins are proteins that associate with and activate CDKs at specific points in the cell cycle. In particular, the D-type cyclins, which are encoded by three closely related genes (cyclins D1, D2, and D3), play a critical role in responding to extracellular mitogenic signals and govern progression through the G_1_ phase via their association with CDK4 and CDK6. Proteasome-mediated degradation serves as a critical mechanism for regulating the steady-state levels of cyclin D1 and ensuring the proper control of cell cycle progression. When cyclin D1 is deregulated, through overexpression, accumulation, or inappropriate localization, it becomes an oncogene and contributes to genomic instability and tumor development (*2*). Aberrant overexpression of cyclin D1 is frequently observed in human cancers (*3*). This deregulation serves as a biomarker of the cancer phenotype and progression of the disease (*4*, *5*). Cyclin D1 and its associated CDKs are promising therapeutic targets in human cancers. However, the precise mechanisms that regulate cyclin D degradation and cellular levels are still not fully understood.

Central to the UPS is the action of ubiquitin E3 ligases, which are enzymes responsible for recognizing specific protein substrates and facilitating the transfer of ubiquitin molecules onto them. Within eukaryotes, the Cullin-RING ligases (CRLs) constitute the largest family of E3 ligases. These ligases are composed of a scaffold protein Cullin and a catalytic RING subunit, RING-box protein 1 (RBX1) or RBX2 (*6*–*8*). The damage-specific DNA binding protein 1 (DDB1) acts as an integral component of the Cullin4A/B (CUL4A/B)–RING E3 ubiquitin ligase (CRL4) complex. It serves as an adaptor protein between CUL4A/B- and DDB1-CUL4-associated factors (DCAFs), facilitating the targeting of substrates for ubiquitination. DDB1 has three β-propeller domains [β-propeller A (BPA), BPB, and BPC] and has the ability to interact with various substrate receptors. These receptors, in turn, recruit specific substrates for further processing.

**Fig. 1.**
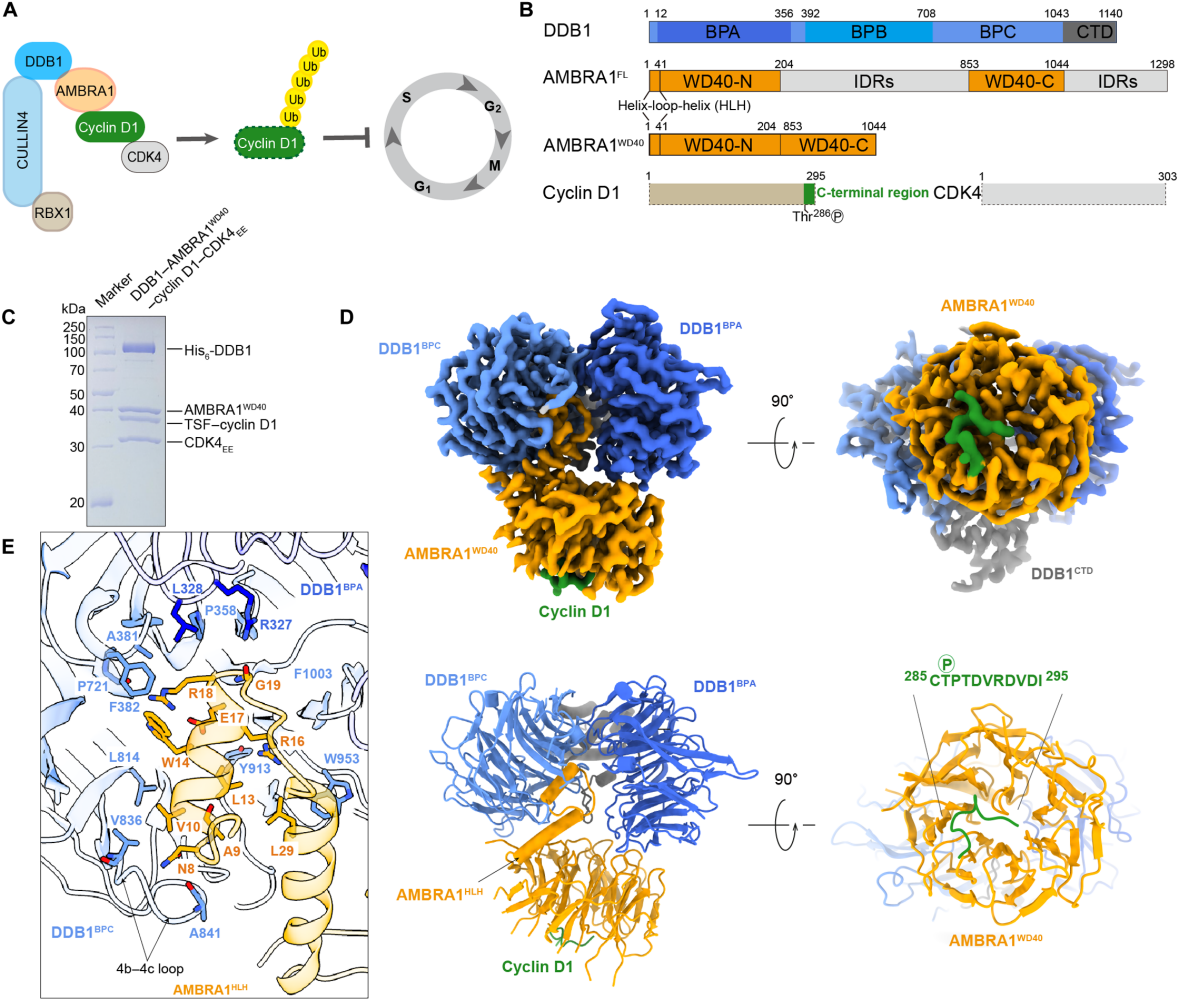
The cryo-EM structure of DDB1–AMBRA1^WD40^–cyclin D1. (**A**) Cartoon schematic of the Cullin4-DDB1-RBX1-AMBRA1 E3 ligase–mediating ubiquitination of cyclin D1 in regulating cell cycle progression. Ub, ubiquitin. (**B**) Annotated DDB1, AMBRA1, cyclin D1, and CDK4 domain schematics. The AMBRA1^WD40^ construct includes the N-terminal helix-loop-helix, WD40-N, and WD40-C. CTD, C-terminal helical domain; FL, full-length; IDRs, intrinsically disordered regions. (**C**) Coomassie blue–stained SDS–polyacrylamide gel electrophoresis (SDS-PAGE) analysis of purified DDB1–AMBRA1^WD40^–cyclin D1–CDK4_EE_ complex used for cryo-EM structure determination. (**D**) The cryo-EM density map and the refined coordinates of the DDB1– AMBRA1^WD40^–cyclin D1 complex. AMBRA1^WD40^ is colored in orange, the BPA domain in dark blue, the BPC-CTD domain in light blue and gray, and the C-terminal region of cyclin D1 in green. (**E**) Close-up view of the AMBRA1^HLH^-DDB1 interface. The key residues contributing to the interface are labeled.

AMBRA1 (autophagy and beclin 1 regulator 1) functions as the E3 ligase receptor involved in the regulation of autophagy and cell cycle control. AMBRA1 acts as a substrate recognition subunit of the CRL4^DDB1^ E3 ligase complex and facilitates the ubiquitination and proteasomal degradation of its target proteins. AMBRA1 functions as the main regulator of the degradation of D-type cyclins (Fig. 1A) (*9*–*11*). AMBRA1 recognizes and ubiquitinates the C-terminal phosphodegron of D-type cyclins that contain a highly conserved threonine at position 286 (*1*, *10*, *12*). Phosphorylation of Thr^286^ by glycogen synthase kinase 3β facilitates the binding of cyclin D1 to the nuclear exportin chromosome region maintenance 1 (CRM1) and triggers cyclin D1 nuclear export and proteolytic turnover (*13*, *14*). Mutation that blocked the phosphorylation of Thr^286^ (T286A) was shown to inhibit cyclin D1 degradation via UPS in mouse fibroblasts (*12*). Similarly, the T286A mutation impaired AMBRA1–cyclin D1 interaction and stabilized cyclin D1 in human cell lines (*10*). Thr^286^ is mutated in a variety of human cancers, and the overexpression of mutant alleles in animal models drives spontaneous tumors (*1*, *15*). AMBRA1 is a tumor suppressor that is mutated in a wide range of human tumors (*16*, *17*). Loss of AMBRA1 and the resulting stabilization of cyclin D1 lead to increased progression of the cell cycle, genomic instability, and decreased sensitivity to CDK4/6 inhibitors (*10*, *11*, *18*, *19*). Therefore, AMBRA1 has emerged as a potential target for anticancer treatment and a biomarker for cancer therapy (*20*).

Recent studies showed that AMBRA1 is a highly dynamic and largely intrinsically disordered protein. AMBRA1 contains a “split” WD40 domain (AMBRA1^WD40^) consisting of two halves separated by a long, intrinsically disordered region (Fig. 1B). These two parts need to reunite to form a seven-bladed β-propeller that binds to DDB1 through the N-terminal helix-loop-helix motif (*18*, *21*). This interaction was shown to be similar to other E3 substrate receptors bound to DDB1, including DCAF1, Cockayne syndrome group A protein, and simian virus 5 V (*18*). We have previously shown that the AMBRA1 WD40 domain is sufficient for cyclin D1 recognition and ubiquitination, highlighting its functional role in substrate recruitment (*18*). However, the molecular mechanism of substrate binding by AMBRA1 is unknown. In addition to cyclin D1, numerous other AMBRA1-interacting proteins have been identified (*22*), allowing AMBRA1 to function as a hub in various physiological processes, including autophagy, development, cell death, and proliferation. Structures of AMBRA1 in complex with substrates are necessary to provide insight into the recognition of diverse target proteins and set a foundation for the development of potential therapeutic strategies.

Here, we report the cryo–electron microscopy (cryo-EM) structure of the human DDB1-AMBRA1^WD40^ E3 ligase receptor complex bound to the cyclin D1–CDK4 complex as a substrate. The structure reveals a protein-protein–interacting surface on AMBRA1^WD40^ that binds to the extreme C terminus of cyclin D1, and this interaction is dependent on phosphorylation at Thr^286^. Experimental structures allowed for the comparison of AlphaFold3 models and in silico analysis of AMBRA1 binding to other D-type cyclins. The in vitro biochemical studies show that the AMBRA1 WD40 domain can bind and mediate the ubiquitination of the cyclin D1 C terminus in a phosphorylation-dependent manner. Consistent with these data, we further show that mutations that disrupt the AMBRA1–cyclin D1 interaction cause endogenous cellular DNA damage. These studies provide important mechanistic insights into the AMBRA1–cyclin D1 recognition and suggest a common mechanism for the AMBRA1-mediated ubiquitination of D-type cyclins in a phosphorylation-dependent manner.

## RESULTS

### Structure of DDB1-AMBRA1^WD40^ bound to cyclin D1–CDK4

To understand AMBRA1 substrate recognition, we determined the structure of the cyclin D1–CDK4 in complex with AMBRA1^WD40^ domain and DDB1 (DDB1-AMBRA1^WD40^) by single-particle cryo-EM at a resolution of 3.55 Å. The protein complex was purified from Expi293F cells by coexpressing the human AMBRA1 WD40 domain with full-length cyclin D1, CDK4_EE_, and DDB1. In the CDK4 construct, amino acids G43 to G47 were replaced with EE (CDK4_EE_) to increase protein stability (*23*). AMBRA1^WD40^ construct was generated as described previously and comprised residues M1 to N204, directly fused to residues S853 to G1044 (*18*). The purified DDB1–AMBRA1^WD40^–cyclin D1–CDK4_EE_ complex appeared as a stable heterotetramer judged by SDS–polyacrylamide gel electrophoresis (SDS-PAGE) and size exclusion chromatography (Fig. 1C and fig. S1A). Cryo-EM images were collected and processed as detailed in Materials and Methods (fig. S2). Two-dimensional (2D) classification and 3D refinement procedures converged to the part of the complex containing DDB1 and AMBRA1^WD40^. The resulting electron density map allowed unambiguous tracing of the AMBRA1^WD40^ domain, the BPA and BPC domains of DDB1, and the extreme C terminus of cyclin D1, allowing us to build a reliable atomic model (Fig. 1D). Posttranslational modification analysis including Western blotting and mass spectrometry (MS) of the reconstituted protein complex, which was used for structural determination, revealed the presence of Thr^286^ phosphorylation in cyclin D1 (fig. S1, H and I).

The overall structure of the DDB1-AMBRA1^WD40^ receptor subcomplex agrees well with our previously reported structure without the substrate (*18*), with the map and model quality substantially improved. The interaction between AMBRA1 and DDB1 is mediated by the helix-loop-helix motif located at the N terminus of AMBRA1 (residues E6 to K41). This motif inserts in the binding pocket formed between the BPA and BPC double propeller of DDB1 (Fig. 1, D and E). The side chain of N8 and the backbone NH of A9, located at the N-terminal end of the first helix of AMBRA1, form hydrogen bonds with the backbone CO groups in the loop connecting β strands b and c in the blade IV of the DDB1 BPC domain. The side chains of residues A9, V10, L13, and W14 in AMBRA1 contribute to the interactions by packing against the surface of the DDB1 BPC domain. At the C-terminal end of the first helix, the side chains of residues R16 and R18 form multiple hydrogen bonds with DDB1. R16 forms hydrogen bonds with Y913 of DDB1, while the guanidinium group of R18 interacts with the side chain of E17 in AMBRA1 and the backbone carbonyl group of P721 in DDB1. The side-chain methylene groups of R18 and the side chain of W14 are accommodated by a hydrophobic patch on the BPC domain formed by L328, P358, A381, and F382. Last, R327 in DDB1 forms a hydrogen bond with the backbone carbonyl group of G19 (Fig. 1E). This interaction interface is further strengthened by the second helix of AMBRA1, which packs against helix 1 and also interacts with the surface of the DDB1 BPC domain (Fig. 1E). The Cullin binding domain (BPB) of DDB1 exhibits high conformational heterogeneity and was subtracted during data processing to improve the resolution of the final reconstruction (fig. S2).

**Fig. 2.**
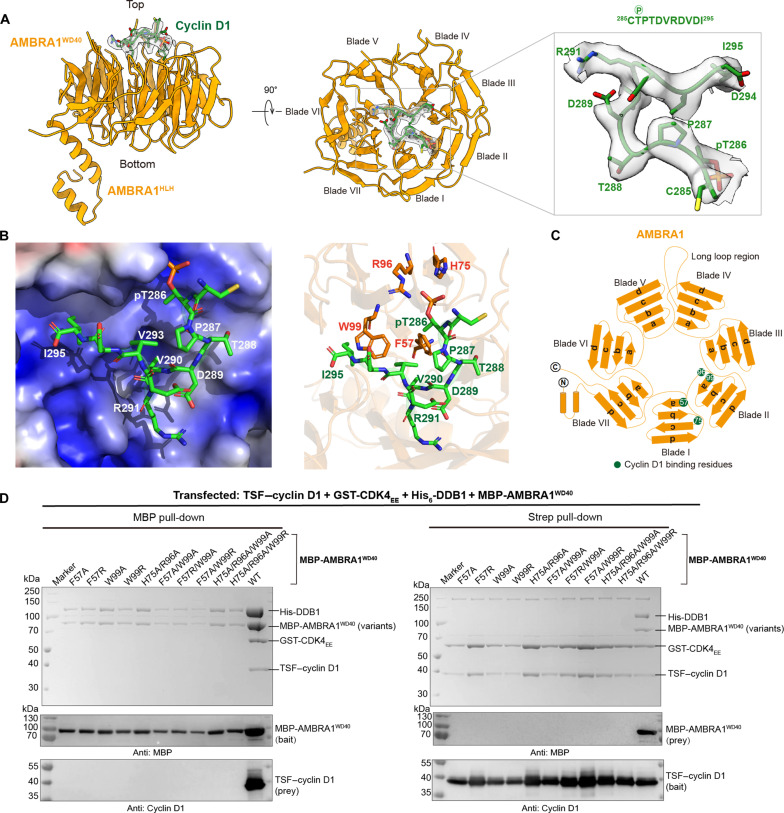
The interface of the AMBRA1^WD40^–cyclin D1 complex. (**A**) The model of AMBRA1^WD40^–cyclin D1 complex. The close-up view of map density and the coordinate of cyclin D1 C-terminal region. (**B**) The electrostatic potential of the AMBRA1^WD40^–cyclin D1 complex interface. The key residues contributing to the interaction of AMBRA1^WD40^ are labeled. (**C**) The cartoon of the AMBRA1 and the key binding residues are labeled in green cycle. (**D**) In vitro pull-down experiments of maltose-binding protein (MBP)–AMBRA1^WD40^ or key binding residue mutants with TSF–cyclin D1. The cells were cotransfected with TSF–cyclin D1, GST-CDK4_EE_, His_6_-DDB1, and MBP-AMBRA1^WD40^. The experiment was repeated at least three times and visualized by SDS-PAGE and Western blotting.

Structured regions of AMBRA1 consist of the N-terminal helix-loop-helix motif, followed by the WD40 domain. The AMBRA1^WD40^ domain exhibits a β-propeller architecture, composed of seven blades, and each blade contains four antiparallel β strands (a to d). The AMBRA1^WD40^ β-propeller structure has a wider bottom and a narrower top when observed from the side view and packs against the BPA and BPC domains of DDB1 with a bottom surface (Figs. 1D and 2A). One distinctive structural feature of AMBRA1 is the presence of the 650-residue intrinsically disordered region in blade IV, between the b and c β strands. This split WD40 domain organization of AMBRA1 is stabilized by DDB1 and proposed to facilitate the interaction between diverse substrates and CRL4 E3 ligase, thereby aiding in the transfer of ubiquitin (*18*). The density for most of the cyclin D1–CDK4_EE_ subcomplex was not visible, probably due to its flexibility, and only the last 11 residues of cyclin D1 assume well-defined conformation in the electron density map. From the overall structure, the extreme C-terminal region of cyclin D1 (residues C285 to I295) binds in a turn-like conformation to the top surface of AMBRA1^WD40^ and covers the central pore of the β-propeller (Figs. 1D and 2A). The binding of the substrate to the exposed top surface of AMBRA1^WD40^ orients the substrate for subsequent ubiquitination by the E3 ligase complex.

### Molecular interactions of AMBRA1^WD40^ with cyclin D1

The specificity of the phosphorylation-dependent recognition of cyclin D1 can be entirely attributed to the structural features present within the WD40 domain of AMBRA1 and includes hydrophobic, hydrogen bonding, and electrostatic interactions. The interaction interface between cyclin D1 C terminus and AMBRA1^WD40^ spans across blades I and II and extends to the center of the β-propeller. The pThr^286^ (phosphorylated Thr^286^) phosphate group interacts with the side chains of R96 and H75 and forms hydrogen bond with the backbone NH of T97 in AMBRA1^WD40^ (Fig. 2, B and C). The residues that follow the phosphorylation site adopt a turn-like conformation, which is immediately followed by a single-turn 3_10_ helix formed by residues D289 to D292. This structure tightly packs against the top surface of AMBRA1^WD40^ through nonpolar interactions between the side chains of residues V290, V293, and I295 in cyclin D1 and a hydrophobic pocket flanked by the side chains of residues F57 and W99 in AMBRA1^WD40^. The C-terminal carboxylate group of I295 in cyclin D1 is stabilized by positive charges that outline the cyclin D1–binding site in AMBRA1^WD40^ (Fig. 2B). There are no large structural rearrangements in AMBRA1^WD40^ upon binding of cyclin D1, based on a root mean square deviation (RMSD) of 1.322 Å (fig. S3A). Together, cyclin D1 recognition by the WD40 domain of AMBRA1 is determined by several factors, including a specific binding site for pThr, a hydrophobic binding pocket that preferentially accommodates nonpolar residues positioned downstream of the phosphorylation site, and an electrostatic surface potential that favors acidic residues/functional groups.

The key cyclin D1–binding residues in AMBRA1^WD40^ were tested by mutagenesis. The hydrophobic residues were mutated to alanine or arginine, and the positively charged amino acids were replaced with alanine. The interaction between cyclin D1 and AMBRA1^WD40^ was tested by pull-down analysis. We coexpressed the Twin-Strep-FLAG (TSF)–cyclin D1, glutathione *S*-transferase (GST)–CDK4_EE_, His-DDB1, and maltose-binding protein (MBP)–AMBRA1^WD40^ variants harboring mutations in the pThr^286^ binding site and the hydrophobic pocket, including F57A, F57R, W99A, W99R, H75A/R96A, F57A/W99A, F57R/W99A, F57A/W99R, H75A/R96A/W99A, and H75A/R96A/W99R. The MBP pull-down demonstrated that none of the AMBRA1^WD40^ mutants was able to bind cyclin D1. Similar results were shown in the Strep pull-down, and only cyclin D1 was able to bind to the wild-type (WT) AMBRA1^WD40^ (Fig. 2D). 2D class averages of purified AMBRA1 F57A, W99R, and H75A/R96A mutants in complex with DDB1 showed the entirety of the AMBRA1^WD40^ domain and are similar to the WT protein, indicating that point mutations did not cause protein misfolding or influence interaction with DDB1 (fig. S4). In summary, the pull-down experiments confirmed that the AMBRA1^WD40^ interacts with cyclin D1 C terminus through the residues F57, H75, R96, and W99 and that both the pT268-binding site and the hydrophobic pocket are essential for the intermolecular interaction.

**Fig. 3.**
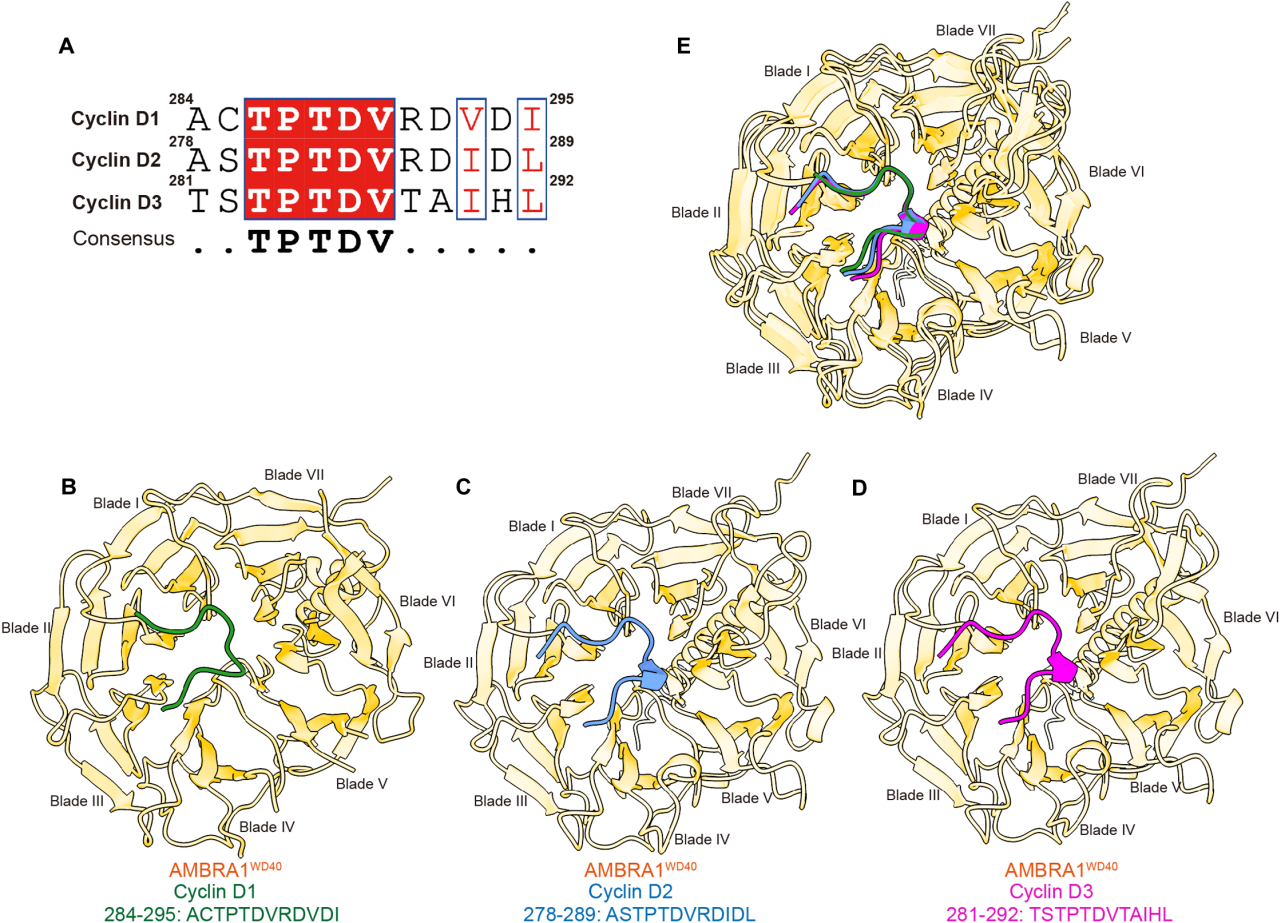
Comparison of D-type cyclins in complex with AMBRA1^WD40^. (**A**) The sequence alignment of the D-type cyclins. Cyclin D1 peptides used for alignment are residues A284 to I295 resolved in cryo-EM structure and its corresponding cyclin D2 residues A278 to L289 and cyclin D3 residues T281 to L292. Residues conserved among D-type cyclins are highlighted in red. (**B**) The experimental model AMBRA1^WD40^–cyclin D1. (**C**) AlphaFold3-predicted model of AMBRA1^WD40^–cyclin D2 and (**D**) AMBRA1^WD40^–cyclin D3. (**E**) The overlap of the D-type cyclins and AMBRA1^WD40^ complex.

Earlier studies have shown that CRL4^AMBRA1^ complex can target cyclins D1, D2, and D3 for proteasome-mediated degradation (*24*). All three D-type cyclins exhibit a high degree of sequence and domain conservation, including the C-terminal threonine phosphodegron site (*1*, *19*). Thr^286^ and the downstream hydrophobic amino acids that directly interact with AMBRA1^WD40^ are conserved in cyclins D2 and D3 (Fig. 3A). An AlphaFold3-predicted (*25*) model of the human AMBRA1^WD40^–cyclin D1 complex agrees well with our experimental structure with an RMSD of 0.324 Å for the cyclin D1 C terminus (fig. S3, B to D). This result prompted us to perform an in silico analysis of AMBRA1 interaction with the phosphodegron sequences of cyclins D2 and D3. The AlphaFold3-predicted models show that the C termini of cyclins D2 and D3 adopt the same turn-like conformation, which is followed by a 3_10_ helix, and bind to the top surface of AMBRA1^WD40^ in a manner nearly identical to cyclin D1 (Fig. 3, B to E). pThr interaction mode with AMBRA1^WD40^ is identical in all three models. Therefore, the broad structural features described here are consistent with the AMBRA1 role as a major regulator of cyclin D levels in cells and underscore the key role of phosphorylation in regulating cyclin D recognition and degradation via the ubiquitin-proteasome pathway.

### AMBRA1^WD40^ recognizes the C-terminal phosphodegron of D-type cyclins and mediates their ubiquitination

**Fig. 4.**
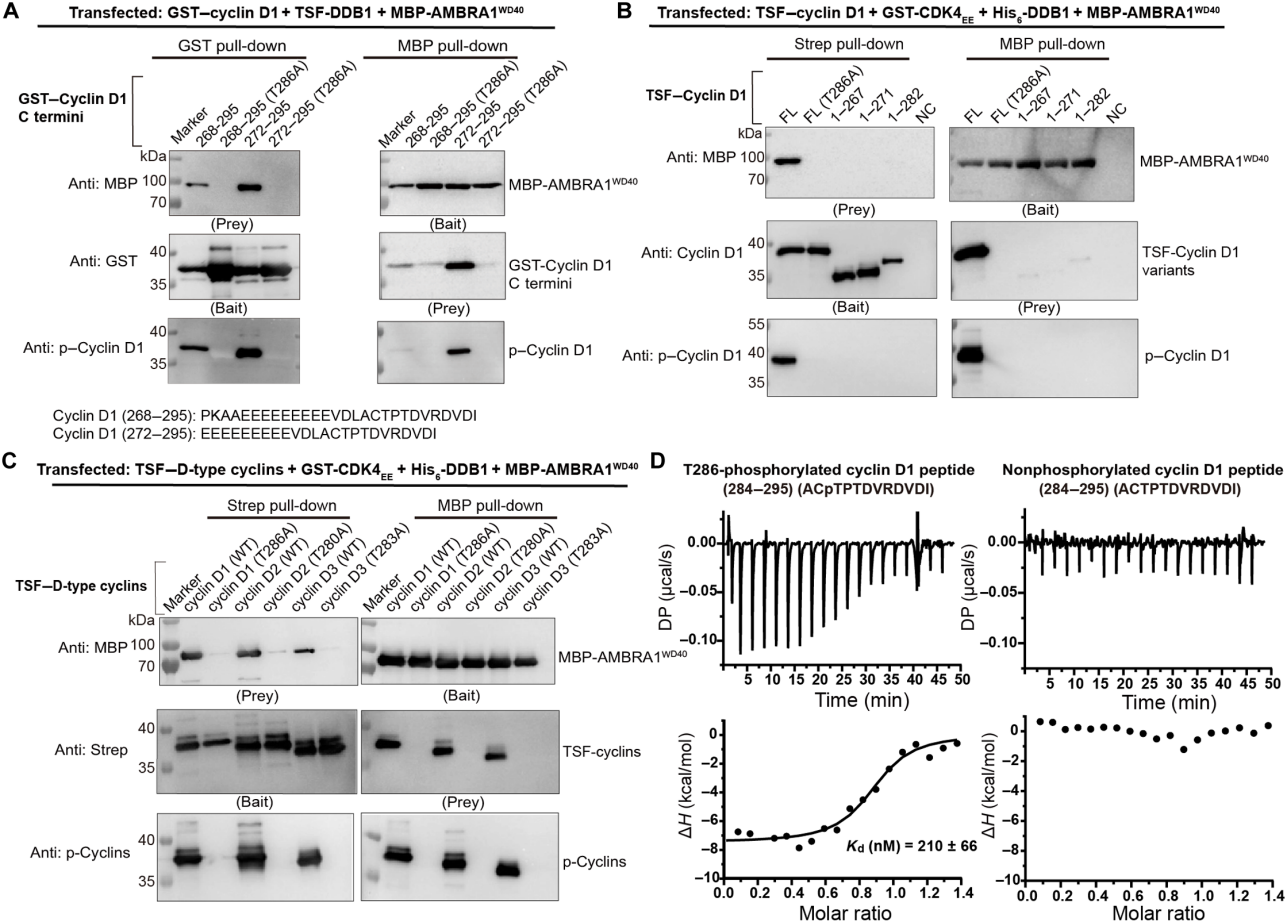
AMBRA1^WD40^ is capable to bind D-type cyclins. (**A**) In vitro pull-down experiment of MBP-AMBRA1^WD40^ with GST-tagged C-terminal region of cyclin D1 and T286A mutants. The experiment was repeated at least three times and visualized by Western blotting. p–Cyclin D1, phosphorylated cyclin D1. (**B**) In vitro pull-down experiment of MBP-AMBRA1^WD40^ with cyclin D1 C-terminal truncations and T286A mutants. The cells were cotransfected with TSF–cyclin D1, GST-CDK4_EE_, His_6_-DDB1, and MBP-AMBRA1^WD40^. NC, negative control. For strep pull-down, NC was only transfected with His_6_-DDB1 and MBP-AMBRA1^WD40^. For MBP pull-down, NC was only transfected with TSF-cyclin D1 and GST-CDK4_EE_. The experiment was repeated at least three times and visualized by Western blotting. (**C**) In vitro pull-down experiment of MBP-AMBRA1^WD40^ with cyclins D1, D2, D3, D1^T286A^, D2^T280A^, and D3^T283A^ mutants. The cells were cotransfected with TSF–D-type cyclins, GST-CDK4_EE_, His_6_-DDB1, and MBP-AMBRA1^WD40^. The experiment was repeated at least three times and visualized by Western blotting. (**D**) The binding affinity of T286-phosphorylated and nonphosphorylated cyclin D1 C-terminal peptide (residues 284 to 295) and the DDB1-AMBRA1^WD40^ complex were detected by ITC. The *K*_d_ value of the T286-phosphorylated cyclin D1 C-terminal peptide and DDB1-AMBRA1^WD40^ complex was 210 ± 66 nM, and the nonphosphorylated cyclin D1 C-terminal peptide was not capable of binding DDB1-AMBRA1^WD40^ complex. DP, differential power.

**Fig. 5.**
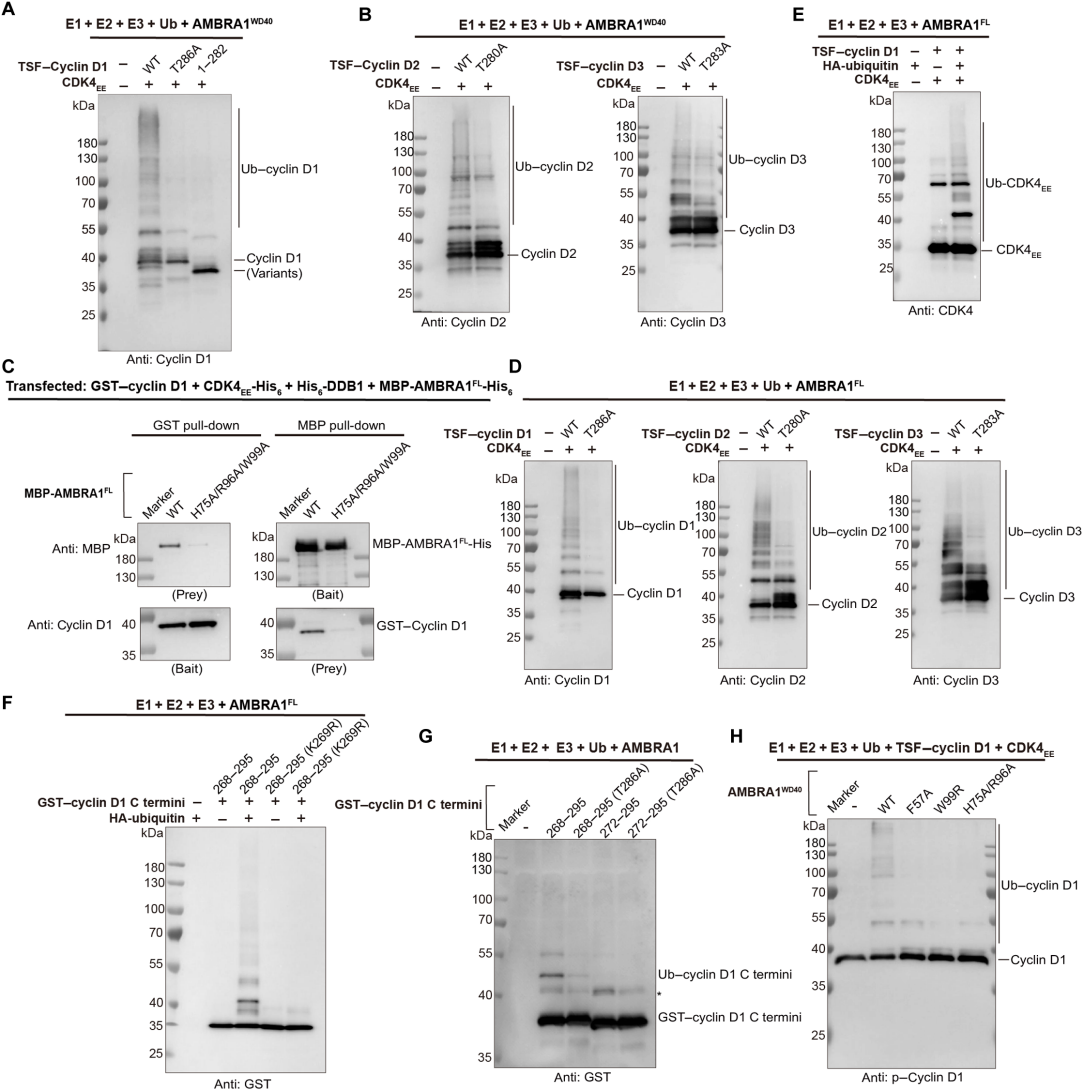
AMBRA1 mediates the ubiquitination of D-type cyclins. (**A**) Ubiquitination assay of AMBRA1^WD40^ mediated the polyubiquitination of TSF–cyclin D1, T286A mutant, and C-terminal truncation. The experiment was repeated independently three times with similar results. (**B**) Ubiquitination assay of AMBRA1^WD40^ mediated the polyubiquitination of TSF–cyclin D2/cyclin D2^T280A^ and TSF–cyclin D3/cyclin D3^T283A^. The experiment was repeated independently three times with similar results. (**C**) In vitro pull-down experiment of GST–cyclin D1 with MBP-AMBRA1^FL^-His and H75A/R96A/W99A mutant. The cells were cotransfected with GST–cyclin D1, CDK4_EE_-His_6_, His_6_-DDB1, and MBP-AMBRA1^FL^-His or MBP-AMBRA1^H75A/R96A/W99A^-His mutant. The experiment was repeated at least three times and visualized by Western blotting. (**D**) Ubiquitination assay of AMBRA1^FL^ mediated the polyubiquitination of TSF–D-type cyclins and threonine phosphorylation site mutants. The experiment was repeated independently three times with similar results. (**E**) Ubiquitination assay of AMBRA1^FL^ mediated the polyubiquitination of CDK4 was detected. The experiment was repeated independently three times with similar results. (**F**) Ubiquitination assay of AMBRA1^FL^ mediated the polyubiquitination of GST–cyclin D1 C terminus, the hemagglutinin (HA)–ubiquitin, and K269R mutant as negative control. The experiment was repeated independently three times with similar results. (**G**) Ubiquitination assay of AMBRA1 mediated the polyubiquitination of GST–cyclin D1 C termini. Asterisk indicates the nonspecific band. The experiment was repeated independently three times with similar results. (**H**) Ubiquitination assay of AMBRA1^WD40^ WT and key binding site mutants mediated the polyubiquitination of cyclin D1. The results were analyzed by Western blotting. The experiment was repeated independently three times with similar results.

To address whether cyclin D1 C terminus is sufficient to bind AMBRA1^WD40^, we performed a series of pull-down assays. Cyclin D1 residues P268 to I295 were directly fused to GST and coexpressed with CDK4_EE_-His, TSF-DDB1, and MBP-AMBRA1^WD40^. Using GST–cyclin D1 (residues P268 to I295) construct as bait was sufficient to pull down AMBRA1^WD40^. The same result was obtained using MBP-AMBRA1^WD40^ as bait, showing that the cyclin D1 C-terminal sequence is sufficient for recognition by AMBRA1^WD40^. The Western blotting analysis using anti–p–cyclin D1 antibody confirmed the presence of T286 phosphorylation in all pulled samples (Fig. 4A). We therefore tested the requirement for T286 phosphorylation using T286A mutants. T286A mutation largely decreased the interaction with AMBRA1^WD40^, indicating phosphorylation-dependent interaction (Fig. 4A). Subsequently, we tested whether cyclin D1 C terminus is necessary for the interaction. For this, we cotransfected the three truncated TSF–cyclin D1 constructs (residues M1 to D267, M1 to A271, or M1 to D282) individually with GST-CDK4_EE_, His-DDB1, and MBP-AMBRA1^WD40^. The C-terminal truncations almost completely abolished the interaction with AMBRA1^WD40^, indicating that the cyclin D1 C terminus is as well necessary for the interaction with AMBRA1^WD40^ (Fig. 4B). Next, we performed pull-down assays using full-length cyclins D1, D2, and D3. All three D-type cyclins were able to associate with AMBRA1^WD40^, while the corresponding threonine phosphorylation site mutations (cyclin D1 T286A, cyclin D2 T280A, and cyclin D3 T283A) almost completely abolished AMBRA1^WD40^ binding (Fig. 4C). Further isothermal titration calorimetry (ITC)–based analyses revealed that the T286-phosphorylated cyclin D1 peptide (residues 284 to 295) can directly bind to the DDB1-AMBRA1^WD40^ complex with a dissociation constant (*K*_d_) value of ∼200 nM, while the nonphosphorylated peptide did not interact (Fig. 4D). Therefore, the C-terminal phosphodegron of D-type cyclins is the key region responsible for phosphorylation-dependent interaction with the AMBRA1 WD40 domain. To examine whether AMBRA1^WD40^ can mediate the ubiquitination of D-type cyclins, we performed in vitro ubiquitination assays using TSF–D-type cyclins and the corresponding threonine phosphorylation site mutants. Consistent with the pull-down results, all three D-type cyclins were polyubiquitinated when incubated with the CRL4 ligase complex (Fig. 5, A and B), and the threonine phosphorylation site mutants showed reduced ubiquitination activity in all three D-type cyclins (Fig. 5, A and B). Furthermore, the full-length AMBRA1 (AMBRA1^FL^) was also used to perform the pull-down and in vitro ubiquitination assays. We coexpressed the GST–cyclin D1, CDK4_EE_-His, His-DDB1, and MBP-AMBRA1-His or MBP-AMBRA1(H75A/R96A/W99A)-His mutant. Pull-down assays reveal that these mutations nearly completely disrupt the interaction between cyclin D1 and AMBRA1, indicating that the WD40 domain serves as the primary binding interface for cyclin D1 (Fig. 5C). While some residual binding may arise from interactions with the AMBRA1 intrinsically disordered regions, these regions do not appear to be essential for cyclin D1 binding. In vitro ubiquitination assays using AMBRA1^FL^ showed similar trends as observed for the AMBRA1^WD40^ (Fig. 5D), indicating that the AMBRA1 WD40 domain is sufficient to mediate the ubiquitination of D-type cyclins. Furthermore, the ubiquitination of CDK4 was detected in the assay, suggesting that CDK4 undergoes bystander ubiquitination during the AMBRA1-mediated process (Fig. 5E).

Previous studies have shown that residue K269, which is located in the proximity of the C-terminal phosphodegron, is essential for the ubiquitination of cyclin D1 by the SKP1/cullin/F-box protein (SCF) ligase, resulting in its degradation (*26*). Similarly, more recent posttranslational modification analysis suggests that the T286 plays an important regulatory role in controlling the ubiquitination of cyclin D1 at the K269, as the major site for this modification (*10*). To address whether AMBRA1^WD40^ can mediate the ubiquitination of the cyclin D1 C terminus at K269, we performed in vitro ubiquitination assays using GST–cyclin D1 (residues P268 to I295) and GST–cyclin D1 (residues E272 to I295) constructs. Both constructs were able to associate with AMBRA1^WD40^ (Fig. 4A); however, only the construct that includes K269 [GST–cyclin D1 (residues P268 to I295)] showed robust ubiquitination when incubated with the CRL4 ligase complex. Furthermore, a K269R mutant construct of identical length failed to undergo ubiquitination, showing the specificity of K269 as the ubiquitination site (Fig. 5F). Consistent with the previous studies, the corresponding T286A mutant showed strongly reduced ubiquitination activity (Fig. 5G). To address whether AMBRA1^WD40^ F57A, W99R, and H75A/R96A mutants influence cyclin D1 ubiquitination, we purified the AMBRA1^WD40^ mutants in complex with Cullin4A-DDB1-RBX1 E3 ligase and CDK4_EE_–cyclin D1 complex and performed in vitro ubiquitination assays (fig. S1, B and C). AMBRA1^WD40^ is sufficient to promote the ubiquitination of cyclin D1 if every enzyme of the ubiquitination cascade reaction is included (Fig. 5, A and H). However, the polyubiquitination of cyclin D1 was strongly decreased in the presence of all tested AMBRA1^WD40^ mutants when compared with the WT protein, which is consistent with the pull-down results (Figs. 2D and 5H). Together, our results imply that AMBRA1^WD40^ is sufficient for phosphorylation-dependent association with the cyclin D1 C terminus and subsequent ubiquitination at K269.

### Mutations that disrupt the AMBRA1–cyclin D1 interaction cause DNA damage

**Fig. 6.**
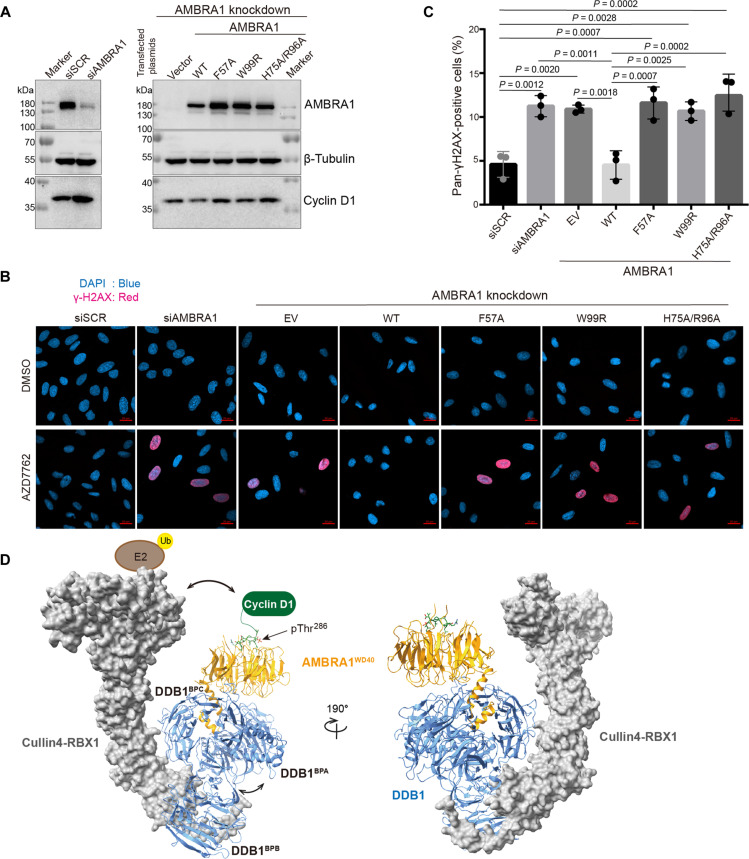
AMBRA1 mutants result in increased sensitivity to CHK1 inhibition. (**A**) AMBRA1 was knocked down by specific siRNA in U2OS cells. Then, cells were transfected with the empty vector, AMBRA1 WT, and mutants. The protein expression level was analyzed by Western blotting, and β-tubulin was used as the protein loading control. The experiment was repeated independently three times with similar results. siSCR, scrambled siRNA. siAMBRA1, AMBRA1 siRNA. (**B**) The representative images of pan-γH2AX–positive cells after AMBRA1 knockdown, AMBRA1 WT, and mutant overexpression. Cells were treated with dimethyl sulfoxide (DMSO) or 100 nM AZD7762. EV, empty vector. Imaging was collected using a Zeiss LSM 900 confocal microscope. 4′,6-Diamidino-2-phenylindole (DAPI) was shown in blue, and γH2AX was shown in red. Scale bars, 20 μm. (**C**) Quantification of Pan-γH2AX–positive cells. For each group, around 300 cells were manually counted using ZEN software, and the percentage of pan-γH2AX–positive cells was calculated. EV, empty vector. Data were plotted in GraphPad Prism 9.4.1 software. Statistical significance was determined by Tukey’s multiple comparisons of one-way analysis of variance (ANOVA), and dots indicate individual data points for *n* = 3 biological replicates. *P* values are indicated in the graph. Graph bar was presented as mean values ± SD. (**D**) Hypothetic model of Cullin4–RBX1–DDB1–AMBRA1^WD40^–cyclin D1 complex. The AMBRA1^WD40^–cyclin D1 structure in this study aligned to Cullin4A-DDB1-RBX1 structures (Protein Data Bank: 2HYE). Cullin4 and RBX1 are presented on the surface, and DDB1, AMBRA1, and cyclin D1 are colored in blue, orange, and green, respectively. The pThr^286^ is indicated with the arrow. The movement of DDB1 between BPA and BPC with BPB facilitates the E2 close to a substrate that allows the ubiquitination of cyclin D1.

We further examined the effect of the interaction between cyclin D1 and AMBRA1 on the DNA damage in U2OS cells. It was reported that AMBRA1 depletion leads to an increase in cellular proliferation, S-phase enrichment, replication stress, and DNA damage, which is greatly intensified by the inhibition of checkpoint kinase 1 (CHK1) (*11*). Small interfering RNA (siRNA) oligoribonucleotides were used to knock down endogenous AMBRA1 in U2OS cells (Fig. 6A). Consistent with previous studies, we found that AMBRA1 down-regulation results in the higher expression levels of cyclin D1 and also significantly increased DNA damage in cells treated with CHK1 inhibitor AZD7762 (Fig. 6, A and B). We have investigated the effect of overexpression of the WT and mutated AMBRA1 on endogenous DNA damage in AMBRA1-silenced cells. Re-expression of WT AMBRA1 was sufficient to rescue the noninterfered cell phenotype and reduce the cyclin D1 expression level (Fig. 6, A to C). All tested AMBRA1 mutants failed to attenuate the high levels of endogenous DNA damage and down-regulate the cyclin D1 expression level (Fig. 6, A to C). In summary, F57, W99, H75, and R96 are the key binding sites, which are essential for the AMBRA1-mediated ubiquitination and degradation of cyclin D1, thereby directly affecting the essential downstream cellular functions. These results highlight the critical role of AMBRA1 in the regulation of cyclin D1 protein level in cells and demonstrate the importance of the AMBRA1 in preventing replication stress and genome instability.

## DISCUSSION

The overexpression and amplification of D-type cyclins are frequently found in several types of human cancer (*1*). Emerging evidence indicates that AMBRA1 is a major regulator of the stability of D-type cyclins, including cyclins D1, D2, and D3, through interaction with DDB1 and CRL4 (*9*–*11*). By promoting their degradation, AMBRA1 helps maintain proper cyclin D levels, preventing overaccumulation that can lead to uncontrolled cell proliferation. Understanding their interaction may provide critical insights into therapeutic targets for cancers with altered cell cycle regulation. Our previous studies have shown that AMBRA1 interacts with the DDB1 double propeller via multiple interfaces, which involve the N-terminal helix-loop-helix motif and the bottom surface of the AMBRA1 WD40 domain (*18*). This bipartite interaction mechanism is accompanied by the stabilization effect on the AMBRA1 split WD40 domain dynamics. In this study, we determined the structure of the DDB1–AMBRA1–cyclin D1 complex and identified the WD40 domain as a crucial structural element used by AMBRA1 to recognize the D-type cyclins. AMBRA1 interactions with most of the binding partners, including Beclin1, dynein light chain 1, microtubule-associated protein 1 light chain 3 (LC3), and protein phosphatase 2A, are mediated by sequences that localize to the intrinsically disorder regions (*17*, *22*, *27*–*29*). Interaction with DDB1 is proposed to stabilize the split WD40 domain in AMBRA1 and bring these regions to the proximity of the CRL4 active site. Our study now shows that AMBRA^WD40^ can directly recognize and bind to a highly conserved extreme C terminus of cyclin D1. Cyclin D1 C terminus folds into a turn-like conformation and tightly packs against the top surface of the AMBRA1 β-propeller, indicating a general mode for the recognition of D-type cyclins. Our results indicate that the complete, fully formed β-propeller assembly of the AMBRA1 WD40 domain is necessary for it to recognize and mediate the ubiquitination of D-type cyclins, and interaction with DDB1 may be an important stabilizing factor.

D-type cyclins, which share about 57% sequence identity, contain a conserved C-terminal phosphodegron that determines their degradation by the UPS (*1*). Using in silico predictions and biochemical assays, we found that AMBRA^WD40^ is likely able to recognize and bind to cyclins D2 and D3 in a manner similar to how it binds to cyclin D1. The structure shows that cyclin D1 residue T286 is phosphorylated in the reconstituted complex and plays a pivotal role in cyclin D1 interaction with the AMBRA1 WD40 domain. Cyclin D1 (T286A) mutation prevents interaction with AMBRA1^WD40^ and impairs ubiquitination near the C terminus of cyclin D1. Our findings agree with previous studies demonstrating that in contrast to the WT cyclin D1, the depletion of AMBRA1 does not induce the accumulation of cyclin D1 (T286A) (*10*). In addition to this key residue, AMBRA1 F57 and W99 residues are lining the hydrophobic ligand binding pocket, which, combined with the T286-interacting residues H75 and R96, complete the cyclin D1 recognition site. Mutations of any of these residues impaired the ability of AMBRA1^WD40^ to interact and mediate the ubiquitination of cyclin D1. Furthermore, none of the mutants was able to rescue the increased DNA damage in the AMBRA1-silenced cells, consistent with cyclin D1 stabilization. These observations underscore the importance of AMBRA1^WD40^–cyclin D1 interaction in regulating cell proliferation, by mediating cyclin D1 degradation, and the prevention of replication stress and DNA damage. The general mode of cyclin D1 recognition is consistent with previous studies showing that the top surface of WD40 domains frequently interacts with proteins that are targeted for ubiquitination [reviewed in (*30*)]. The SCF/FBXO31 ubiquitin ligase complex, which is involved in DNA damage–induced cyclin D1 degradation, is capable of phosphorylation-independent cyclin D1 ubiquitination by recognizing and binding to its extreme C terminus (*31*). In contrast, the recognition mode of AMBRA1–cyclin D1 is phosphorylation dependent, indicating that distinct regulatory pathways govern cyclin D1 turnover in these two complexes. We propose a model in which phosphorylation on Thr^286^ that activates cyclin D1 nuclear export (*13*) is required for its association with AMBRA1. This interaction mode results in cyclin D1 presentation toward the RBX1 binding site of the E2 ubiquitin–conjugating enzyme (Fig. 6D). Neddylation of Cullin likely induces RBX1 to adopt an extended catalytic conformation (*32*), thereby facilitating the transfer of ubiquitin from the E2 enzyme to cyclin D1.

Our structural and biochemical findings uncovered a detailed mechanism of the CRL4^AMBRA1^-mediated degradation of D-type cyclins. The CRL4^AMBRA1^ complex plays a crucial role in targeting D-type cyclins for ubiquitination and subsequent proteasomal degradation. By understanding the molecular interactions and conformational changes that occur during this process, our study sheds light on the broader implications of CRL4^AMBRA1^ activity in cell cycle control. Future studies are needed to determine whether AMBRA1^WD40^ can directly recognize substrates other than cyclins D and the functional role of the intrinsically disordered regions. Small molecular compounds such as molecular glues and PROTACs (proteolysis targeting chimeras) are at the forefront of targeted protein degradation technology. They function by promoting or enhancing the interaction between E3 ubiquitin ligases and target proteins, leading to the ubiquitination and proteasomal degradation of the substrate. Targeting the DDB1–AMBRA1–cyclin D1 complex for cancer therapy offers promising strategies to enhance the degradation of cyclin D1. By enhancing cyclin D1 degradation, it may be possible to sensitize cancer cells to CDK4/6 inhibitors, overcoming resistance and improving therapeutic outcomes. In addition, this approach could inhibit tumor progression by disrupting the cyclin D1–driven proliferation of cancer cells. Structural studies of the AMBRA1 and D-type cyclins have laid the foundation for the development of small molecules targeting the E3 ligase–receptor–substrate complex and provide the potential treatment for diseases characterized by dysregulated cyclin activity, such as cancer.

## MATERIALS AND METHODS

### Antibodies

Anti-AMBRA1 antibody was used at 1:1000 dilution [Cell Signaling Technology (CST), 24907S]; anti–phospho-histone H2A.X (Ser^139^) antibody was used at 1:1000 dilution (CST, 2577S); F(ab′)2 fragment (Alexa Fluor 647 conjugate) was used at 1:1000 dilution (CST, 4414S); anti–cyclin D1 antibody was used at 1:1000 dilution (CST, 2922S, and Santa Cruz, sc-20044); anti–cyclin D2 antibody was used at 1:1000 dilution (Proteintech, 10934-1-AP); anti–cyclin D3 antibody was used at 1:1000 dilution (Proteintech, 26755-1-AP); anti–phospho–cyclin D1 (Thr^286^) antibody was used at 1:2000 dilution (CST, 3300T); anti–phospho–cyclin D2 (Thr^280^) antibody was used at 1:2000 dilution (Abcam, AB230818); anti–phospho–cyclin D3 (Thr^283^) antibody was used at 1:2000 dilution (Affinity, AF3251); anti-CDK4 antibody was used at 1:2000 dilution (Proteintech, 11026-1-AP); anti–β-tubulin antibody was used at 1:1000 dilution (Novus, NB600-936); anti-MBP tag mouse monoclonal antibody was used at 1:1000 dilution (GenScript, A00190); GST tag mouse monoclonal antibody was used at 1:1000 dilution (STARTER, S-372-19); and the secondary antibodies conjugated to horseradish peroxidase were used as follows: Goat anti-Mouse immunoglobulin G (IgG) was used at 1:2000 dilution [JiangSu CoWin Biotech (CWBIO), CW0102], and goat anti-rabbit IgG was used at 1:2000 dilution (CWBIO, CW0103).

### Cloning and mutagenesis

The gene for full-length AMBRA1 (residues M1 to R1298) was codon optimized. The genes encoding DDB1, Cullin4A, RBX1, cyclin D1, CDK4, ubiquitin-like modifier-activating enzyme 3 (UBA3), amyloid precursor protein-binding protein 1 (APPBP1), NEDD8-conjugating enzyme (UBC12), and ubiquitin-like protein (NEDD8) were amplified from cDNA by polymerase chain reaction (PCR) and subcloned into pCAG vectors with different tags using Kpn I and Xho I cutting sites, respectively. AMBRA1^WD40^ (residues M1 to N204/S853 to G1044) was subcloned with an N-terminal MBP tag or a GST tag, followed by a tobacco etch virus (TEV) protease cutting site. For cyclins D1, D2, and D3, all were cloned with an N-terminal TSF tag. DDB1 was constructed with an N-terminal His_6_ tag or an N-terminal TSF tag. Residues G43 to G47 in CDK4 were replaced with Glu-Glu (CDK4_EE_) to increase protein stability (*23*). CDK4_EE_ was constructed with an N-terminal GST tag, followed by a TEV cutting site. Cullin4A and RBX1 were constructed with an N-terminal TSF tag.

WT, truncations, and mutations of cyclin D1 were constructed with an N-terminal TSF tag for the Strep pull-down. The C-terminal region was cloned with an N-terminal GST tag and followed by a (GGGGS)_2_ linker used for GST pull-down.

The AMBRA1^WD40^ mutants were generated by PCR-based site-directed mutagenesis approaches. For AMBRA1^WD40^ WT, AMBRA1^WD40^ F57A, F57R, W99A, W99R, H75A/R96A, F57A/W99A, F57R/W99A, F57A/W99R, H75A/R96A/W99A, and H75A/R96A/W99R used in the MBP pull-down experiment, they were cloned as an N-terminal MBP tag. For coexpression with Cullin4A-DDB1-RBX1 E3 ligase, AMBRA1^WD40^ WT, AMBRA1^WD40^ F57A, W99R, and H75A/R96A were constructed with an N-terminal GST tag. For in vivo cell experiments, tagless AMBRA1^FL^, AMBRA1^F57A^, AMBRA1^W99R^, and AMBRA1^H75A/R96A^ were cloned into the pCAG vector. The primers used in this study are listed in table S1.

### Protein expression and purification

For mammalian cell expression, MBP-AMBRA1^WD40^, His_6_-DDB1, TSF–cyclin D1, and GST-CDK4_EE_ were cotransfected and purified for structural studies. GST-AMBRA1^WD40^ (WT and mutants), TSF-Cullin4A, TSF-DDB1, and TSF-RBX1 were cotransfected and purified for in vitro ubiquitination assay. TSF–cyclin D1 (WT and mutants) and GST-CDK4_EE_ were coexpressed for in vitro ubiquitination assay. MBP-AMBRA1^WD40^ (WT and mutants) and His_6_-DDB1 were coexpressed for negative staining.

The Expi293F cells were grown in Union-293 medium and used for protein expression, with 1 mg of DNA and 4 mg of polyethyleneimine (PEI) per 1 liter of cells at a density of 2 × 10^6^ cells/ml. Cells were harvested after transfected for 3 days and washed with 1× cold phosphate-buffered saline (PBS).

For AMBRA1^WD40^, DDB1, cyclin D1, and CDK4_EE_ coexpression, the cell pellet was lysed in lysis buffer A [50 mM Hepes (pH 7.4), 200 mM NaCl, 10% (v/v) glycerol, 1% (v/v) Triton X-100, 2 mM MgCl_2_, and 5 mM β-mercaptoethanol] with protease inhibitors [1 mM phenylmethylsulfonyl fluoride (PMSF), 0.15 μM aprotinin, 10 μM leupeptin, and 1 μM pepstain] for 30 min at 4°C. After centrifugation at 39,191*g* for 30 min, MBP beads were incubated with the cell supernatant at 4°C for 2 hours. The beads were washed with 20 bed volumes of wash buffer B [50 mM Hepes (pH 7.4), 200 mM NaCl, 2 mM MgCl_2_, and 5 mM β-mercaptoethanol]. Bound proteins were eluted with wash buffer B containing 10 mM maltose. After TEV digestion overnight, the elution was then subjected to Strep-tactin sepharose resin, washed with 20 bed volumes of wash buffer B, and eluted with gel filtration buffer C [50 mM Hepes (pH 7.4), 200 mM NaCl, 2 mM MgCl_2_, and 1 mM Tris-(2-Carboxyethyl)phosphine hydrochloride (TCEP)] supplemented with 5 mM desthiobiotin. The protein elution was further purified by Superdex 200 Increase 10/300 GL (Cytiva). The sample was used for cryo-EM structure analysis. The SDS-PAGE for the purified proteins was shown in Fig. 1C, and the gel filtration profile was shown in fig. S1A.

The AMBRA1^WD40^ (WT and mutants) and Cullin4A-DDB1-RBX1 E3 ligase complex were purified as previously described (*18*). Briefly, the cell pellet was lysed in lysis buffer A with protease inhibitors (1 mM PMSF, 0.15 μM aprotinin, 10 μM leupeptin, and 1 μM pepstain) for 30 min at 4°C. After centrifugation at 39,191*g* for 30 min, GST beads were incubated with the cell supernatant at 4°C for 2 hours. The beads were washed with 20 bed volumes of wash buffer B and eluted in wash buffer B containing 50 mM reduced glutathione. After TEV digestion overnight, the elution was further applied to Strep-tactin sepharose resin, washed with 20 bed volumes, and eluted with gel filtration buffer C containing 5 mM desthiobiotin. The purified proteins are shown in fig. S1B.

For cyclin D1/D2/D3 (WT and mutants)–CDK4_EE_ complex, the cell pellet was lysed in lysis buffer A with protease inhibitors (1 mM PMSF, 0.15 μM aprotinin, 10 μM leupeptin, and 1 μM pepstain) for 30 min at 4°C. After centrifugation at 39,191*g* for 30 min, GST beads were incubated with the cell supernatant at 4°C for 2 hours. The beads were washed with 20 bed volumes of wash buffer B and eluted in wash buffer B containing 50 mM reduced glutathione. After TEV digestion overnight, the elution was further applied to Strep-tactin sepharose resin, washed with 20 bed volumes, and eluted with gel filtration buffer C containing 5 mM desthiobiotin. The SDS-PAGE for the purified proteins was shown in fig. S1 (C and G). GST–linker–cyclin D1 C-terminal proteins were purified by one-step GST affinity purification (fig. S1F). The eluted proteins were further used for in vitro ubiquitination activity assay.

For AMBRA1^WD40^-DDB1 complex, the cell pellet was lysed in lysis buffer A with protease inhibitors for 30 min at 4°C. After centrifugation at 39,191*g* for 30 min, MBP beads were incubated with the cell supernatant at 4°C for 2 hours. The beads were washed with 20 bed volumes of wash buffer B and eluted in wash buffer B containing 10 mM maltose. After TEV digestion overnight, the elution was concentrated and injected into Superdex 200 Increase 10/300 GL (Cytiva) (fig. S1, D and E). The purified proteins were further used for negative staining.

### Negative stain EM imaging

Four microliters of purified protein (0.03 mg/ml) was loaded onto a freshly glow-discharged carbon-coated 300 mesh grid (EMCN, BZ11023a) for 1 min. The grids were subsequently blotted with filter paper and negatively stained with 2% (w/v) uranyl acetate for 40 s. Redundant liquid was absorbed using filter paper. The sample was imaged using a Talos 120C transmission electron microscope (Thermo Fisher Scientific) performed at 120 kV in low-dose mode and imaged with a Ceta complementary metal-oxide semiconductor camera (Thermo Fisher Scientific). Data were collected at a nominal magnification of ×73,000, corresponding to a pixel size of 1.96 Å. The micrographs were further processed by cryoSPARC4.4.1 (*33*).

### Cryo-EM grid preparation and data acquisition

Human DDB1–AMBRA1^WD40^–cyclin D1–CDK4_EE_ complex was prepared for cryo-EM data acquisition. The protein complex was eluted from the peak of the size exclusion column. For cryo-EM grid, 3 μl of protein sample (0.3 mg/ml) was deposited onto freshly glow-discharged Quantifoil R1.2/1.3 Cu300 mesh grids and plunged into liquid ethane using a FEI Vitrobot Mark IV after blotting for 3 s with blot force 0 at 4°C and 100% humidity. A total of 6120 movies were collected on a Titan Krios electron microscope operating at 300 kV equipped with a Gatan K3 camera at a defocus of −1.0 to −1.8 μm in counting mode, corresponding to a pixel size of 0.85 Å. Automated image acquisition was performed using SerialEM with a 3 by 3 image shift pattern. Movies consists of 50 frames, with a total dose of 59.9 e^−^/Å^2^, a total exposure time of 2.5 s, and a dose rate of 17.32 e^−^ per pixel per second. Imaging parameters for the dataset are summarized in table S2.

### Cryo-EM data processing

The movies were divided into five-by-five patches for motion correction by Relion’s own implementation of the MotionCor2 algorithm and then imported into cryoSPARC v4.4.1 (*33*–*36*). Contrast transfer function (CTF) fitting and estimation were performed by patch CTF estimation. After particle picking from an initial subset of 500 micrographs with a blob picker, 322,822 particles were extracted and subjected to iterative rounds of 2D classifications. About 3.7 million particles were picked from the remaining 5620 micrographs. All the picked particles were extracted with a box size of 256 × 256 pixels and further binned to 128 × 128 pixels to speed up the data processing. After iterative 2D classifications, the cleaned 2,794,307 particles were further subjected ab initio to generate four different classes and heterogeneous refinement. The particles from the best three classes were reextracted with a box size of 256 × 256 pixels and chosen for homogeneous refinement. Because of the flexibility of the BPB domain of DDB1, we performed particle subtraction to remove the signal of this domain from particle images, followed by local refinement. After cycles of 2D classifications and one more round of heterogeneous refinement, the best-resolved class was chosen for nonuniform refinement. The final map obtained from the 1,015,935 particles was estimated at 3.55 Å. The map was postprocessed by deepEMhancer with tight target modes and used for model building (*37*). All reported resolutions are based on the gold-standard fourier shell correlation (FSC) 0.143 criterion.

### Atomic model building and refinement

The coordinates for DDB1-AMBRA1^WD40^ (8WQR) were rigid body fitted into the deepEMhancer postprocessed map using UCSF Chimera, and the residues of cyclin D1 were manually built using Coot (*38*). Atomic coordinates were refined by iteratively performing Phenix real-space refinement using the unsharpened map (*39*). Manual inspection and correction of the refined coordinates were performed in Coot (*38*). Model quality was assessed using MolProbity and the map-versus-model FSC by comparing the map-versus-model FSC with the FSC of the experimental cryo-EM density (fig. S5). Figures were prepared using PyMOL 2.3.3 (The PyMOL Molecular Graphics System, version 2.3.3, Schrödinger LLC), UCSF Chimera version 1.15, and ChimeraX version 1.3 (*40*). The cryo-EM density map has been deposited in the Electron Microscopy Data Bank under accession codes EMD-60925 and EMD-63847, and the coordinate has been deposited in the Protein Data Bank under accession number 9IVD.

### Neddylation of Cullin4A with HA-tagged NEDD8

Neddylation of Cullin4A was performed by coexpressing CRL4^AMBRA1 FL/WD40^ with tagless APPBP1, UBA3, UBC12, and hemagglutinin (HA)–tagged NEDD8. For CRL4^AMBRA1 FL^ neddylation, MBP-AMBRA1-His_6_ was cotransfected with Cullin4A-DDB1-RBX1 E3 ligase complex, tagless APPBP1, UBA3, UBC12, and HA-tagged NEDD8 and then purified as previously described AMBRA1^FL^-DDB1-Cullin4A-RBX1 complex purification (*18*). For CRL4^AMBRA1 WD40^ neddylation, GST-AMBRA1^WD40^ was cotransfected with Cullin4A-DDB1-RBX1 E3 ligase complex, tagless APPBP1, UBA3, UBC12, and HA-tagged NEDD8 and then purified as previously described AMBRA1^WD40^-DDB1-Cullin4A-RBX1 complex purification (*18*). Neddylation detection was performed by Western blotting; the purified proteins were further used for cyclin D in vitro ubiquitination assay (Fig. 4, B, F, and G). The SDS-PAGE and Western blotting for the purified proteins were shown in fig. S6.

### In vitro ubiquitination assay

The ubiquitination assays were performed in a 25-μl reaction volume with the following components: 100 nM ubiquitin-like modifier-activating enzyme 1 (UBE1) (Boston Biochem, E-304), 1.5 μM ubiquitin-conjugating enzyme H5C (UBCH5C) (Boston Biochem, E2-627), 0.3 μM purified AMBRA1^WD40^-DDB1-Cullin4-RBX1 or AMBRA1^FL^-DDB1-Cullin4-RBX1 complex, 20 μM HA-ubiquitin (Boston Biochem, U-110), 0.16 μM TSF–D-type cyclins–CDK4_EE_ complex or GST–cyclin D1 C termini, and 10 mM magnesium-ATP solution (Boston Biochem, B-20) in E3 ligase reaction buffer (Boston Biochem, B-71). The reaction was incubated at 37°C for 2 hours and analyzed by SDS-PAGE, followed by immunoblot. The experiment was repeated three times with similar results.

### RNA interference

siRNA oligoribonucleotides corresponding to the human AMBRA1 were ordered from Tsingke. The primers were as follows: AMBRA1 siRNA 1: 5′-GAGUAGAACUGCCGGAUAG-3′; AMBRA1 siRNA 2: 5′-CCACCCAUGUGAACCAUAA-3′; AMBRA1 siRNA 3: 5′-GC-GGAGACAUGUCAGUAUC-3′; AMBRA1 siRNA4: 5′-CUGAAU-CGCUGUCGUGCUU-3′. For siRNA transfection, the human osteosarcoma U2OS cells were plated at 7 × 10^5^ cells per well in a six-well plate. Each well was transfected with the optimal amount of siRNA mixture using Lipofectamine RNAiMAX (Thermo Fisher Scientific) the next day, according to the manufacturer’s instructions.

### Immunoblotting and immunofluorescence

The U2OS cells were plated into a six-well plate and then followed by siRNA transfection using Lipofectamine RNAiMAX (Thermo Fisher Scientific, 13778075) the next day, according to the manufacturer’s instructions. After siRNA transfection for 24 hours, cells were plated into a 12-well plate for transient plasmid transfections the next day. The transient plasmid transfections were performed using X-tremeGENE HP DNA Transfection Reagent (Roche, 06366236001) according to the manufacturer’s instructions. Cells were harvested 48 hours after plasmid transfection. Cells were lysed in lysis buffer [50 mM tris (pH 7.4), 150 mM NaCl, 0.5 mM EDTA, 1% NP-40, and 0.1% SDS] supplemented with protease inhibitors (1 mM PMSF, 0.15 μM aprotinin, 10 μM leupeptin, and 1 μM pepstain) for 15 min on ice and then centrifuged at 15,000*g* for 15 min to remove insoluble debris. Solubilized proteins were quantified by the BCA Protein Assay Kit (Sangon Biotech, C503021), and equal amounts of protein were mixed with loading buffer and heated at 95°C for 5 min. Samples were loaded at the same amount by SDS-PAGE and transferred to 0.22-μm Immuno-Blot polyvinylidene difluoride membranes (Bio-Rad). Membranes were then blocked in 5% milk/TBST buffer (Tris-buffered saline with Tween 20) for 1 hour at room temperature and incubated with primary antibodies at 4°C overnight. For the detection of proteins, using the secondary antibodies conjugated to horseradish peroxidase (anti-mouse and antirabbit; CWBIO) at 1:2000 dilution in TBST for 1 hour at room temperature and visualizing with the cECL Western Blotting Kit (CWBIO, CW0048). The images were acquired by the Amersham Imager 680.

For immunofluorescence, U2OS cells were plated into the 12-well plate on coverslips at 5 × 10^4^ density per well and then followed the same procedure as for immunoblotting. For CHK1 inhibition, cells were treated with 100 nM AZD7762 (MedChemExpress, HY-10992) for 24 hours after plasmid transfection. Cells were washed with cold PBS and fixed in 4% paraformaldehyde (Sangon Biotech, E672002) in PBS for 20 min at room temperature, then permeabilized with PBS/0.1% (v/v) Triton X-100 for 20 min, and subjected to blocking with PBS/1% (w/v) bovine serum albumin (BSA) to block the cell for 1 hour at room temperature. The γH2AX antibody was diluted in PBS/0.01% (v/v) Triton X-100 at 1:1000 dilution and applied to the coverslips at 4°C overnight. The Alexa Fluor 647–conjugated secondary antibody was diluted in PBS/1% (w/v) BSA at 1:1000 dilution and incubated with the coverslips for 1 hour at room temperature. Slides were mounted in an antifade mounting medium with 4′,6-diamidino-2-phenylindole (Beyotime, P0131). Imaging was performed using a Zeiss LSM 900 confocal microscope with a Plan Apochromat 10×/0.95 and 40×/0.95 objective.

### In vitro pull-down and Western blotting experiment

MBP-AMBRA1^WD40^ (WT and mutants), His_6_-DDB1, TSF–cyclin D1/TSF–cyclin D2/TSF–cyclin D3, and GST-CDK4_EE_ were cotransfected into Expi293F cells. The cells were harvested after 3 days of transfection and washed with 1× PBS. After lysis and centrifugation, the supernatant was divided into halves and incubated with MBP or Strep-tactin sepharose resin for 2 hours. The beads were washed twice with lysis buffer A and twice with wash buffer B and further eluted with gel filtration buffer C with 10 mM maltose or 5 mM desthiobiotin. The eluted samples were detected by SDS-PAGE and Western blotting with antibodies against MBP tag and cyclin D1.

To test whether the C-terminal region of cyclin D1 interacts with AMBRA1^WD40^, GST–linker–cyclin D1 (C termini), MBP-AMBRA1^WD40^, TSF-DDB1, and CDK4_EE_-His_6_ were cotransfected into Expi293F cells. The GST, Strep pull-down assay, and Western blotting were performed using the same protocol.

To test whether the C-terminal truncation of cyclin D1 interacts with AMBRA1^WD40^, TSF–cyclin D1 (WT, T286A, 1-282, 1-271, and 1-267), MBP-AMBRA1^WD40^, His_6_-DDB1, and GST-CDK4_EE_ were cotransfected into Expi293F cells. The MBP, Strep pull-down assay, and Western blotting were performed using the same protocol.

To test whether the cyclin D1 interact with full-length AMBRA1 and its mutant, GST–cyclin D1, MBP-AMBRA1 (WT, H75A/R96A/W99A)-His_6_, His_6_-DDB1, and CDK4_EE_-His_6_ were cotransfected into Expi293F cells. The MBP, GST pull-down assay, and Western blotting were performed using the same protocol. The experiment was repeated three times independently.

### Identification of phosphorylation sites by MS

The purified protein complex was digested on an immobilized pepsin column, and eluted peptides were desalted using a trap column (1 mm by 15 mm; Acclaim PepMap300 C18, 5 μm, Thermo Fisher Scientific) for 5 min at a flow rate of 200 μl/min, with a mobile phase consisting of 0.1% formic acid. Subsequent peptide separation was performed on the ACQUITY BEH C18 (2.1 mm by 50 mm) analytical column with a first gradient ranging from 9 to 45% of buffer B (80% acetonitrile and 0.1% formic acid) for 10 min, followed by a second gradient ranging from 45 to 99% of buffer B for 1 min, at a flow rate of 50 μl/min. Peptides were directly ionized via electrospray ionization and analyzed by an Orbitrap Eclipse (Thermo Fisher Scientific) mass spectrometer. A full MS scan was collected in the Orbitrap [mass/charge ratio (*m/z*): 375 to 1500, resolution: 60,000, automatic gain control (AGC) 4e5, and max injection time: 118 ms], followed by a series of tandem MS (MS/MS) scans measured in the Orbitrap (higher-energy collisional dissociation with a normalized collision energy of 30%, resolution: 30,000, AGC 5e4, isolation width: 1.6 *m/z*, and max injection time: 54 ms). Unknown and singly charged ions were excluded from fragmentation. All MS/MS spectra were searched against the custom database containing the sequences of the DDB1, AMBRA1^WD40^, cyclin D1, and CDK4 and common contaminant proteins [CRAPome/Strep tag (*41*)] using the Proteome Discoverer 2.5 software (Thermo Fisher Scientific). The following parameters were used: MS tolerance, 10 parts per million; MS2 tolerance, 0.02 Da; missed cleavages, 2; no enzyme (unspecific); variable modifications, oxidation methionine (+15.995 Da), phosphorylation (S, T, Y, +79.966), and N-terminal acetylation (+42.011 Da). The Percolator node within the Proteome Discoverer was used to filter the peptide spectral match false discovery rate to 1% (strict). The ptmRS node was used for the localization of modification sites within validated peptide sequences (*42*).

### Isothermal titration calorimetry

The cyclin D1–derived peptides used in this study were chemically synthesized by GenScript Biotech, Nanjing, China, with N-terminal acetylation modification and high-performance liquid chromatography–verified purity exceeding 95%. Two distinct peptide variants were used: the cyclin D1 peptide (284 to 295; ACTPTDVRDVDI) and its phosphorylated counterpart containing phosphothreonine at position 286 (ACpTPTDVRDVDI), both prepared at 100 μM working concentration in gel filtration buffer [50 mM Hepes (pH 7.4), 200 mM NaCl, 2 mM MgCl_2_, and 1 mM TCEP]. ITC measurements were performed using a MicroCal PEAQ-ITC system (Malvern Panalytical, UK), and these peptides were titrated into AMBRA1^WD40^-DDB1 complex (10 μM). The experiments were performed under 25°C with constant agitation at 750 rpm. A multi-injection protocol was implemented consisting of an initial 0.4-μl injection, followed by 18 subsequent 2-μl injections, with 150-s intervals between injections to ensure complete thermal equilibration. Reference measurements were acquired through control titrations of peptide solutions into buffer alone, enabling subsequent subtraction of dilution heat effects from the experimental datasets. Data were analyzed using the MicroCal PEAQ-ITC analysis software using a single-site binding model to obtain the *K*_d_, binding enthalpy (Δ*H*), and stoichiometry (*N*).

### Quantification and statistical analyses

For pan-γH2AX–positive cell quantification, a minimum of three independent experiments was included in the representing graphs. For each group, a total of around 300 cells were manually counted using ZEN (Blue edition) software, and pan-γH2AX–positive cell percentage was calculated. Data were analyzed using GraphPad Prism 9.4.1 software. Statistical significance was determined by Tukey’s multiple comparisons of one-way analysis of variance (ANOVA), *P* values are indicated in the graph, and the value of *P* < 0.05 was considered statistically significant. The graph bar was presented as mean values ± SD.
