# Supplementary material for: Mechanism of D-type cyclin recognition by the AMBRA1 E3 ligase receptor
Source: Sci Adv. 2025 May 23;11(21):eadu8708. doi: 10.1126/sciadv.adu8708 (PMC12101500; doi:10.1126/sciadv.adu8708)
Supplement: Supplementary file 1 — Figs. S1 to S6 Tables S1 and S2 [file sciadv.adu8708_sm.pdf]

Science Advances
AAAS

Supplementary Materials for

Mechanism of D-type cyclin recognition by the AMBRA1 E3 ligase receptor

Yang Wang *et al.*

Corresponding author: Ming-Yuan Su, sumy@sustech.edu.cn; Goran Stjepanovic, goranstjepanovic@cuhk.edu.cn

Sci. Adv. **11**, eadu8708 (2025)
DOI: 10.1126/sciadv.adu8708

This PDF file includes:

Figs. S1 to S6
Tables S1 and S2

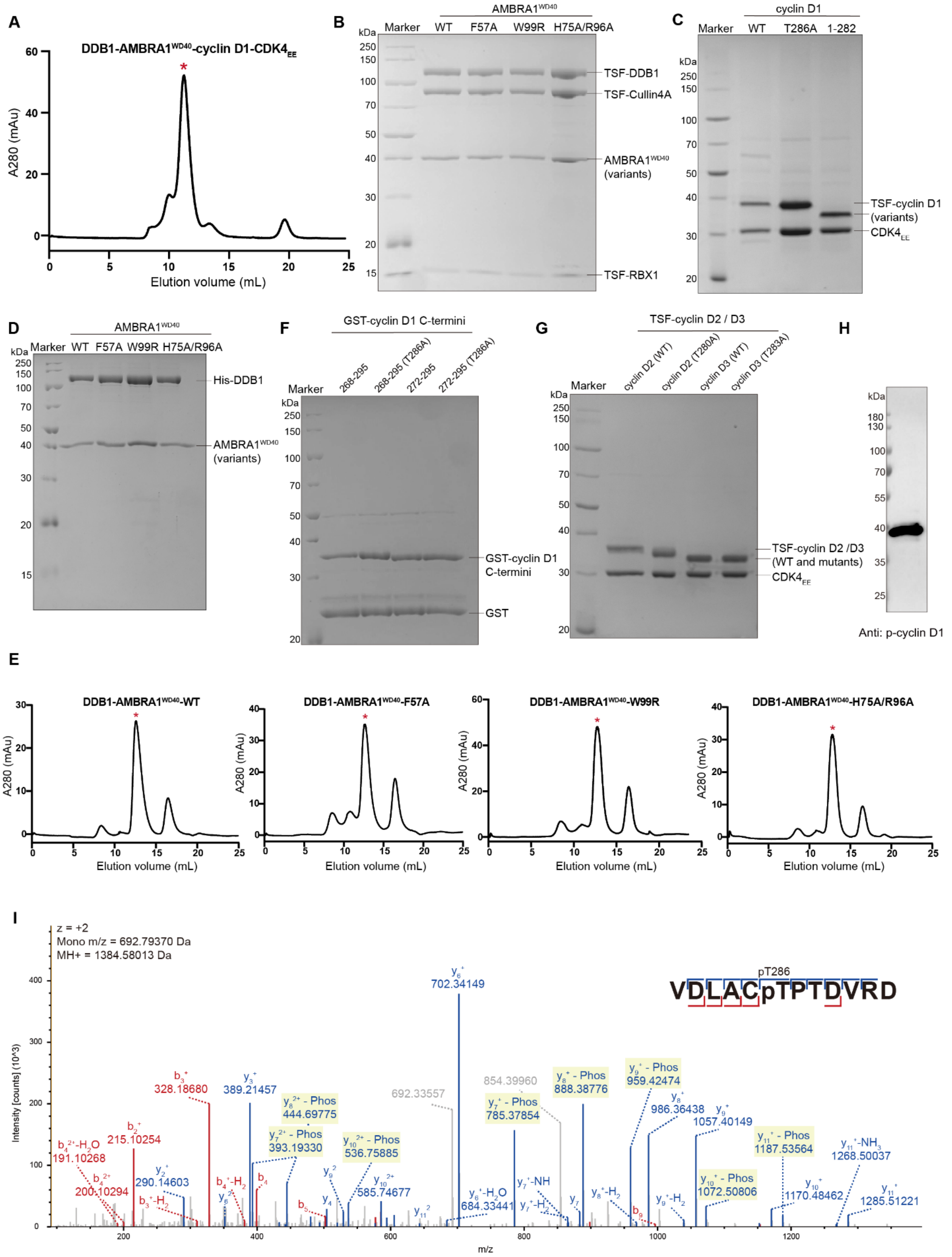
A
DDB1-AMBRA1^{WD40}-cyclin D1-CDK4$_{EE}$
A280 (mAu)
Elution volume (mL)
B
AMBRA1^{WD40}
Marker WT F57A W99R H75A/R96A
kDa
TSF-DDB1
TSF-Cullin4A
AMBRA1^{WD40} (variants)
TSF-RBX1
C
cyclin D1
Marker WT T286A 1-282
TSF-cyclin D1 (variants)
CDK4$_{EE}$
D
AMBRA1^{WD40}
Marker WT F57A W99R H75A/R96A
His-DDB1
AMBRA1^{WD40} (variants)
F
GST-cyclin D1 C-termini
Marker 268-295 268-295 (T286A) 272-295 272-295 (T286A)
GST-cyclin D1 C-termini
GST
G
TSF-cyclin D2 / D3
Marker cyclin D2 (WT) cyclin D2 (T280A) cyclin D3 (WT) cyclin D3 (T283A)
TSF-cyclin D2 /D3 (WT and mutants)
CDK4$_{EE}$
H
Anti: p-cyclin D1
E
DDB1-AMBRA1^{WD40}-WT
DDB1-AMBRA1^{WD40}-F57A
DDB1-AMBRA1^{WD40}-W99R
DDB1-AMBRA1^{WD40}-H75A/R96A
A280 (mAu)
Elution volume (mL)
I
z = +2
Mono m/z = 692.79370 Da
MH+ = 1384.58013 Da
pT286
VDLACpTPTDVRD
Intensity [counts] (10^3)
m/z
y_6^+ 702.34149
b_3^+ 328.18680
y_3^+ 389.21457
y_8^{2+} - Phos 444.69775
y_7^{2+} - Phos 393.19330
y_{10}^{2+} - Phos 536.75885
b_2^+ 215.10254
b_4^{2+}-H_2O 191.10268
b_4^{2+} 200.10294
y_2^+ 290.14603
y_{10}^{2+} 585.74677
692.33557
854.39960
y_7^+ - Phos 785.37854
y_8^+ - Phos 888.38776
y_9^+ - Phos 959.42474
y_8^+ 986.36438
y_9^+ 1057.40149
y_{11}^+ - Phos 1187.53564
y_{11}^+-NH_3 1268.50037
y_{10}^+ - Phos 1072.50806
y_{10}^+ 1170.48462
y_{11}^+ 1285.51221
y_6^+-H_2O 684.33441

Fig. S1. The SDS-PAGE and gel filtration profile of purified protein.

(A) The gel filtration profile of the DDB1-AMBRA1^{WD40}-cyclin D1-CDK4$_{EE}$, the peak was used for cryo-EM structure analysis.

(B) The SDS-PAGE of purified AMBRA1^{WD40}-WT or mutants in complex with E3 ligase.

(C) The SDS-PAGE of purified cyclin D1 WT, mutant and truncation in complex with CDK4$_{EE}$.

(D) The SDS-PAGE of purified DDB1-AMBRA1^{WD40}-WT or mutants complexes, and corresponded gel filtration profiles (**E**).

(F) The SDS-PAGE of purified GST-cyclin D1 C-termini.

(G) The SDS-PAGE of purified TSF-cyclin D2, TSF-cyclin D3 and mutants in complex with CDK4$_{EE}$.

(H) The phosphorylation of cyclin D1 at Thr286 was analysed by western blotting in the reconstituted DDB1-AMBRA1^{WD40}-cyclin D1-CDK4$_{EE}$ protein complex.

(I) MS/MS spectra for the identified phosphorylation site Thr286 in cyclin D1 from purified DDB1-AMBRA1^{WD40}-cyclin D1-CDK4$_{EE}$ protein complex.

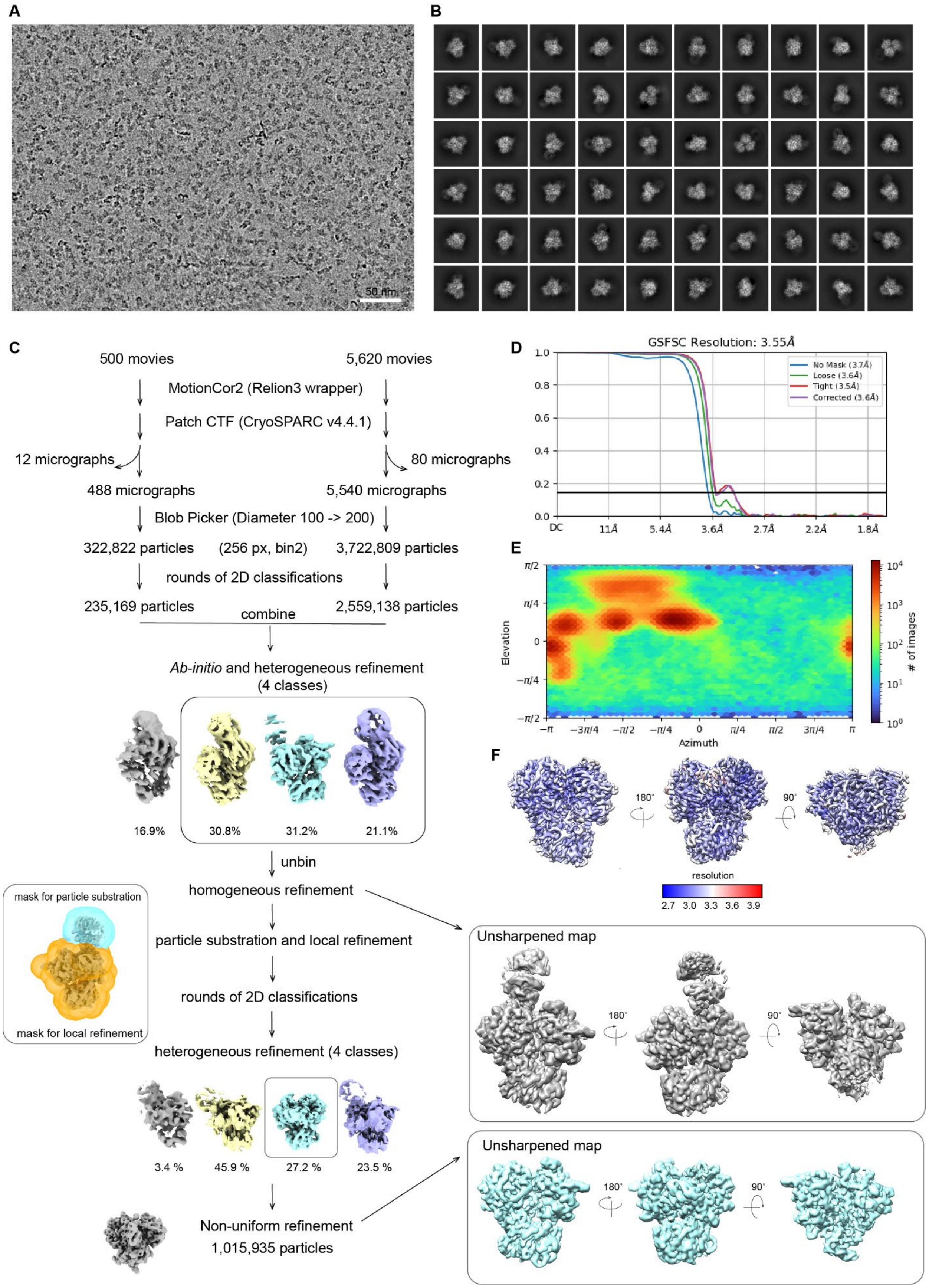
A
50 nm
B
C
500 movies
5,620 movies
MotionCor2 (Relion3 wrapper)
Patch CTF (CryoSPARC v4.4.1)
12 micrographs
80 micrographs
488 micrographs
5,540 micrographs
Blob Picker (Diameter 100 -> 200)
322,822 particles
(256 px, bin2)
3,722,809 particles
rounds of 2D classifications
235,169 particles
2,559,138 particles
combine
Ab-initio and heterogeneous refinement
(4 classes)
16.9%
30.8%
31.2%
21.1%
unbin
homogeneous refinement
mask for particle substration
mask for local refinement
particle substration and local refinement
rounds of 2D classifications
heterogeneous refinement (4 classes)
3.4 %
45.9 %
27.2 %
23.5 %
Non-uniform refinement
1,015,935 particles
D
GSFSC Resolution: 3.55Å
No Mask (3.7Å)
Loose (3.6Å)
Tight (3.5Å)
Corrected (3.6Å)
DC
11Å
5.4Å
3.6Å
2.7Å
2.2Å
1.8Å
E
Elevation
Azimuth
# of images
F
180°
90°
resolution
2.7 3.0 3.3 3.6 3.9
Unsharpened map
180°
90°
Unsharpened map
180°
90°

Fig. S2. Cryo-EM data process of the DDB1-AMBRA1^{WD40}-cyclin D1 complex.

(A) A representative motion-corrected cryo-EM micrograph of the DDB1-AMBRA1 ^{WD40}-cyclin D1 complex. Scale bar equals 50 nm.

(B) Representative 2D class averages for the DDB1-AMBRA1^{WD40}-cyclin D1 complex.

(C) Flow chart of cryo-EM data processing.

(D) The FSC plots are between two independently refined half-maps with no mask (blue), loose mask (green), tight mask (red), and corrected (purple). A cut-off of 0.143 (blue line) was used to estimate the resolution.

(E) Angular particle distribution calculated in cryoSPARC for particle projections. The heatmap shows the number of particles for each viewing angle.

(F) Local resolution is colored as indicated in the scale.

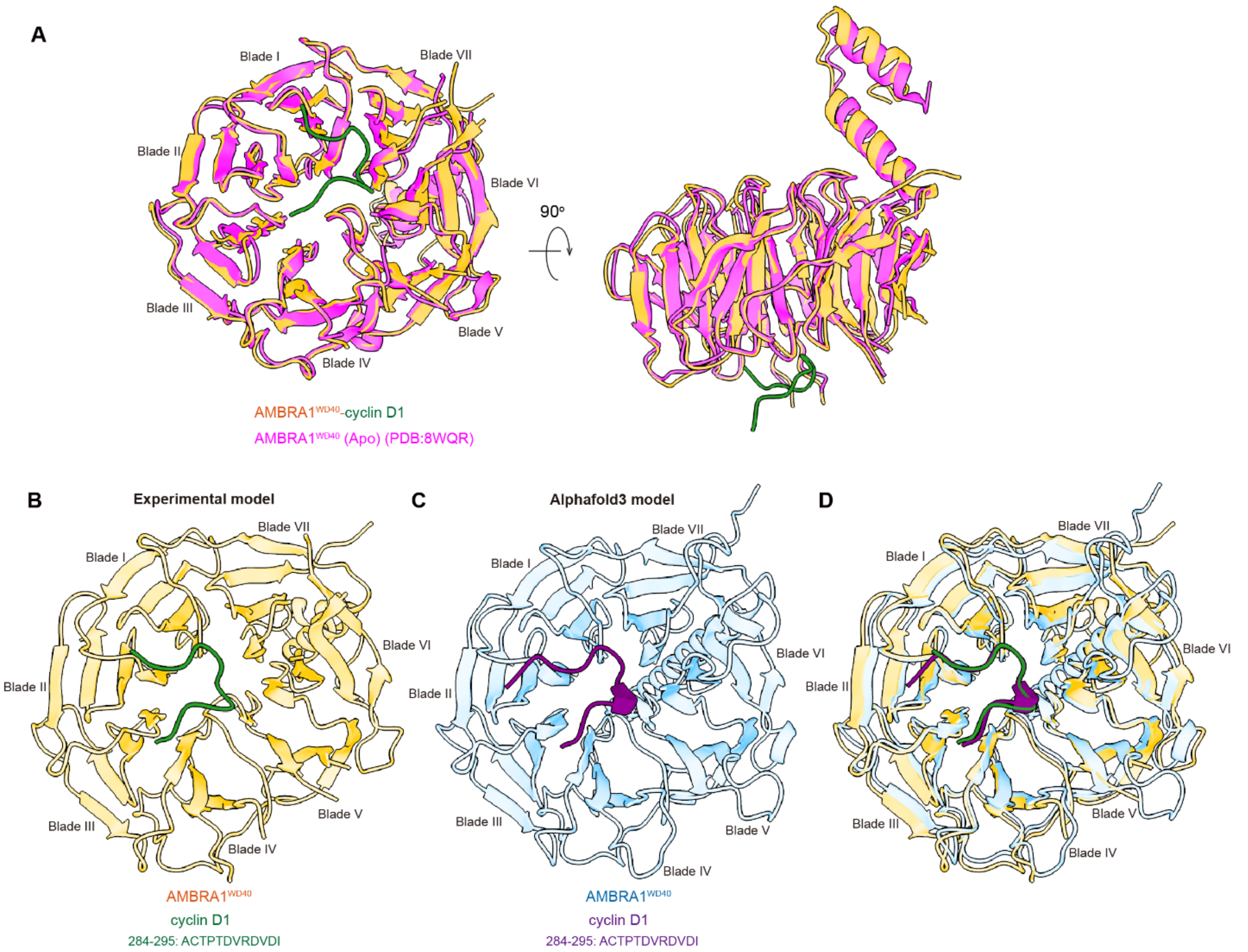

Fig. S3. The comparison between experimental and AlphaFold3 model of AMBRA1^{WD40}-cyclin D1.

(A) The overlay of AMBRA1^{WD40}-cyclin D1 and AMBRA1^{WD40} (Apo) (PDB:8WQR). The overall RMSD value between the AMBRA1^{WD40} models is 1.322 Å. AMBRA1^{WD40} colored in orange, cyclin D1 C-terminus colored in green and AMBRA1^{WD40} (Apo) colored in magenta.

(B) The experimental model of AMBRA1^{WD40}-cyclin D1 C-terminus (284-295). AMBRA1^{WD40} colored in orange and cyclin D1 C-terminus colored in green.

(C) The predicted AlphaFold3 model of AMBRA1^{WD40}-cyclin D1 C-terminus. AMBRA1^{WD40} colored in blue and cyclin D1 C-terminus colored in purple.

(D) The overlay of experimental (**A**) and AlphaFold3 (**B**) model of AMBRA1^{WD40}-cyclin D1. The RMSD value between the cyclin D1 C-terminus of experimental and predicted models is 0.324 Å.

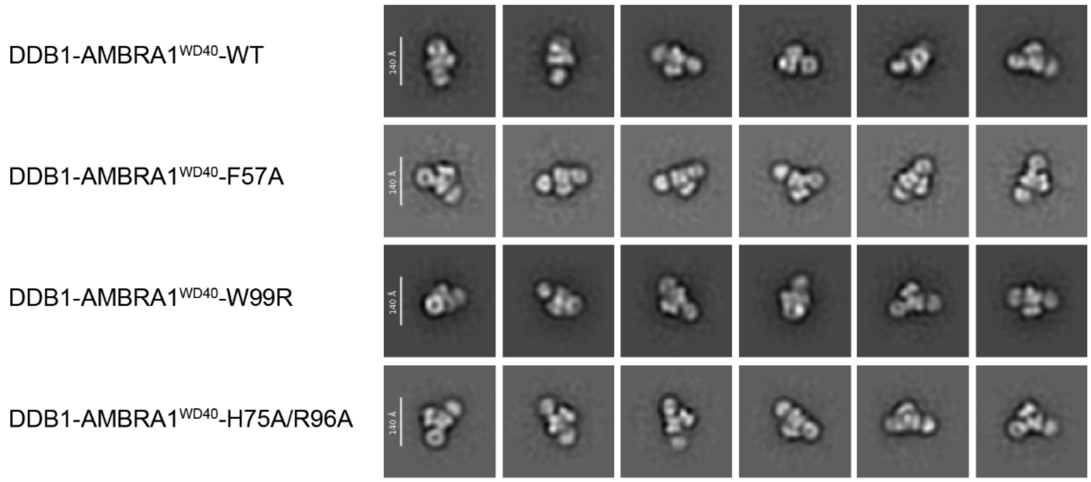

Fig. S4. The representative 2D class averages of DDB1-AMBRA1^{WD40} complex.

The representative 2D class average of negative staining shows the complete WD40 domain of AMBRA1^{WD40} mutants.

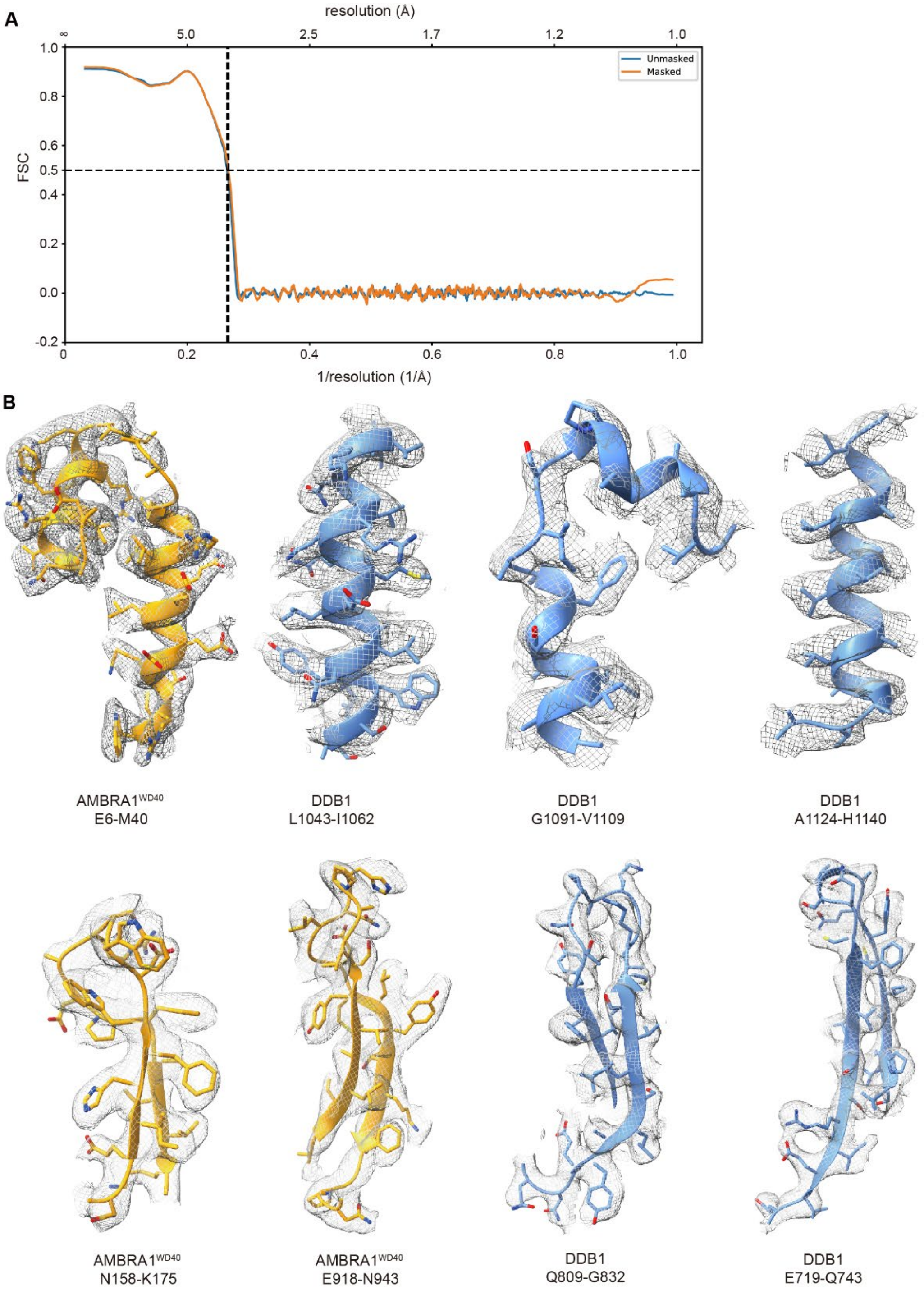

Fig. S5. Model to map fitting.

(A) FSC between the model and map for the DDB1-AMBRA1^{WD40}-cyclin D1 complex against the cryo-EM map.

(B) Representative cryo-EM densities fitted to the model.

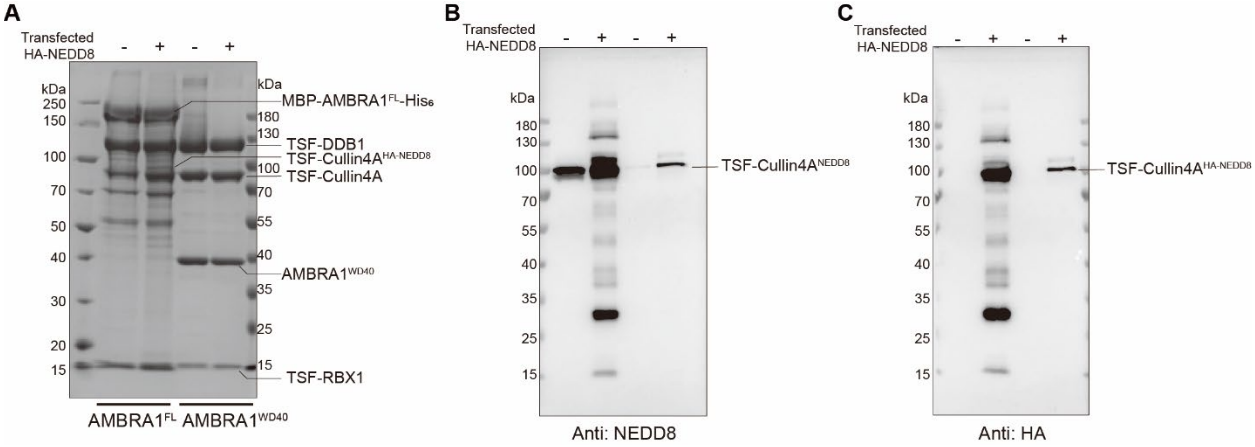

Fig. S6. The SDS-PAGE and western blotting of the purified CRL4$^{AMBRA1\ FL/WD40}$ complex.

(A) The SDS-PAGE of the purified Cullin4A-DDB1-RBX1-AMBRA1$^{WD40/FL}$ complex. The complexes were expressed without and with HA-NEDD8.

(B) The western blotting of purified Cullin4A-DDB1-RBX1-AMBRA1$^{FL/WD40}$ complex corresponding to **(A)**, and detected by anti-NEDD8 antibody **(B)** and anti-HA antibody **(C)**.

Table S1. Primers used in this study.

Construct		Primers (5’ to 3’)
pCAG-GST-CDK4$_{EE}$	Forward primer	CTGTATTTTCAGGGTGGTACCATGGCTACCTCTCGATATG
	Reverse primer	CTGAGGAGTGAATTCCTCGAGTCACTCCGGATTACCTTC
pCAG-TSF-cyclin D1	Forward primer	GACAAGGGTACCATGGAACACCAGCTCCTGTGC
	Reverse primer	GTGAATTCCTCGAGTCAGATGTCCACGTCCCGCAC
pCAG-His$_6$-DDB1	Forward primer	CACCATCACCATCACGGTACCATGTCGTACAACTACGTGG
	Reverse primer	CTGAGGAGTGAATTCCTCGAGCTAATGGATCCGAGTTAGCTC
pCAG-MBP-AMBRA1^{FL}-His$_6$	Forward primer	CAGGGTGGTACCATGAAGGTGGTGCCTGAGAAAAATG
	Reverse primer	GAATTCCTCGAGTCAGTGGTGATGATGGTGATGGCGGTTCCTTGGCTCCCCGCGAG
pCAG-MBP-AMBRA1^{WD40}-WT	Forward primer	CAGGGTGGTACCATGAAGGTGGTGCCTGAGAAAAATG
	Reverse primer	GAATTCCTCGAGCTATCCGCTGTTCAGGGCCTC
pCAG-MBP-AMBRA1$^{(FL\ or\ WD40)}$-F57A	F57A forward primer	GGTCTACCGCCCTGCTGGCTTTTTC
	F57A reverse primer	GAAAAAGCCAGCAGGGCGGTAGACC
pCAG-MBP-AMBRA1$^{(FL\ or\ WD40)}$-W99R	W99R forward primer	GGAGAACCCCCCGGTGTGTGACTTTC
	W99R reverse primer	GAAAGTCACACACCGGGGGGTTCTCC
pCAG-MBP-AMBRA1$^{(FL\ or\ WD40)}$-H75A/R96A	H75A forward primer	CCACGTGAACGCTAATATCTAC
	H75A reverse primer	GTAGATATTAGCGTTCACGTGG
	R96A forward primer	TCGGCCATCGGGCAACCCCCTGGTG
	R96A reverse primer(	CACCAGGGGGTTGCCCGATGGCCGA
pCAG-MBP-AMBRA1^{WD40}-F57R	F57R forward primer	GGTCTACCCGCCTGCTGGCTTTTTC
	F57R reverse primer	GAAAAAGCCAGCAGGCGGGTAGACC
pCAG-MBP-AMBRA1^{WD40}-W99A	W99A forward primer	GGAGAACCCCCGCGTGTGTGACTTTC
	W99A reverse primer	GAAAGTCACACACGCGGGGGTTCTCC
pCAG-MBP-AMBRA1^{WD40}-H75A/R96A/W99A	R96A/W99A forward primer	TCGGCCATCGGGCAACCCCCGCGTGTGTGACTTTC
	R96A/W99A reverse primer	GAAAGTCACACACGCGGGGGTTGCCCGATGGCCGA

pCAG-MBP-AMBRA1^{WD40}-H75A/R96A/W99R	R96A/W99R forward primer	TCGGCCATCGGGCAACCCCCCGGTGTGTGACTTTC
	R96A/W99R reverse primer	GAAAGTCACACACCGGGGGGTTGCCCGATGGCCGA
pCAG-TSF-cyclin D1 (1-282)	Forward primer	GACAAGGGTACCATGGAACACCAGCTCCTGTGC
	Reverse primer	GAATTCCTCGAGTCAGTCCACCTCCTCCTCCTCCT
pCAG-TSF-cyclin D1 (1-271)	Forward primer	GACAAGGGTACCATGGAACACCAGCTCCTGTGC
	Reverse primer	GAATTCCTCGAGTCAGGCGGCCTTGGGGTCCATGT
pCAG-TSF-cyclin D1 (1-267)	Forward primer	GACAAGGGTACCATGGAACACCAGCTCCTGTGC
	Reverse primer	GAATTCCTCGAGTCAGTCCATGTTCTGCTGGGCCTG
pCAG-GST-Linker-cyclin D1 (268-295)	Forward primer	CAGGGTGGTACCCCCAAGGCCGCCGAGGAGGAG
	Reverse primer	TACGCCAAGCTTGGGCTGCAGGTCGAGGGATC
pCAG-GST-Linker-cyclin D1 (272-295)	Forward primer	CAGGGTGGTACCGAGGAGGAGGAAGAGGAGGAG
	Reverse primer	TACGCCAAGCTTGGGCTGCAGGTCGAGGGATC
pCAG-GST-Linker-cyclin D1 (268-295, T286A)	Forward primer	CAGGGTGGTACCCCCAAGGCCGCCGAGGAGGAG
	Reverse primer	GAATTCCTCGAGTCAGATGTCCACGTCCCGCACGTCGGTGGGAGCGCAAGCCAGGTC
pCAG-GST-Linker-cyclin D1 (272-295, T286A)	Forward primer	CAGGGTGGTACCGAGGAGGAGGAAGAGGAGGAG
	Reverse primer	GAATTCCTCGAGTCAGATGTCCACGTCCCGCACGTCGGTGGGAGCGCAAGCCAGGTC
pCAG-GST-Linker-cyclin D1 (268-295,K269R)	Forward primer	CAGGGTGGTACCCCCGGGCCGCCGAGGAGGAG
	Reverse primer	TACGCCAAGCTTGGGCTGCAGGTCGAGGGATC
pCAG-TSF-cyclin D2 (FL)	Forward primer	GATGACGATGACAAGGGTACCATGGAGCTGCTGTGCCACGAG
	Reverse primer	CTGAGGAGTGAATTCCTCGAGTCACAGGTCGATATCCCCGCAC
pCAG-TSF-cyclin D3 (FL)	Forward primer	GATGACGATGACAAGGGTACCATGGAGCTGCTGTGTTGCGAAG
	Reverse primer	CTGAGGAGTGAATTCCTCGAGCTACAGGTGTATGGCTGTGAC

pCAG-TSF-cyclin D2 (FL, T280A)	Forward primer	GATGACGATGACAAGGTACCATGGAGCTGCTGTGCCACGAG
	Reverse primer	CTGAGGAGTGAATTCCTCGAGTCACAGGTCGATATCCCCGCACGTCTGTAGGGGCGCTGGCTTGGTC
pCAG-TSF-cyclin D3 (FL, T283A)	Forward primer	GATGACGATGACAAGGGTACCATGGAGCTGCTGTGTTGCGAAG
	Reverse primer	CTGAGGAGTGAATTCCTCGAGCTACAGGTGTATGGCTGTGACATCTGTAGGGGCGCTGGTCTGGCTGGGCCCTTG
pCAG-MBP-AMBRA1^{FL}-H75A/R96A/W99A-His$_6$	H75A/R96A/W99A forward primer	GAAAATGCGTGCCAGCCTGATCGGCCATCGGGAACCCCCGGAACCCCCGCCGTGTGACTTTCCACCCCTAC
	H75A/R96A/W99A reverse primer	CCGATCAGGCTGTGCACGCACTTTTCCTGTCTTCACCTCGGTAATGTAGATATTGGCGTTCACGTGGGTAG
pCAG-TSF-cyclin D1 (FL, T286A)	Forward primer	GACAAGGGTACCATGGAACACCAGCTCCTGTGC
	Reverse primer	GAATTCCTCGAGTCAGATGTCCACGTCCCGCACGTCGGTGGGAGCGCAAGCCAGGTC

Table S2. Cryo-EM data collection, refinement and validation statistics

	DDB1-AMBRA1^{WD40}-cyclin D1 (EMDB-60925)/ (PDB 9IVD)
**Data collection and processing**	
Magnification	105,000 X
Voltage (kV)	300 kV
Electron exposure ($e^{-}/Å^2$)	59.9
Defocus range (μm)	-1.0 to -1.8
Pixel size (Å)	0.85
Symmetry imposed	C1
Initial particle images (no.)	4,045,631
Final particle images (no.)	1,015,935
Map resolution (Å)<br>FSC threshold	3.55<br>0.143
Map resolution range (Å)	2.86-8.79
**Refinement**	
Initial model used (PDB code)	8WQR (DDB1- AMBRA1^{WD40})
Model resolution (Å)<br>FSC threshold	3.7<br>0.5
Model resolution range (Å)	-
Map sharpening *B* factor ($Å^2$)	-231.1
Model composition<br>Non-hydrogen atoms<br>Protein residues<br>Ligands	 8628<br>1160<br>0
B factors ($Å^2$)<br>Protein (min/max/mean)	 126.48/236.57/164.00
R.m.s. deviations<br>Bond lengths (Å)<br>Bond angles (°)	 0.004<br>0.697
Validation<br>MolProbity score<br>Clashscore<br>Poor rotamers (%)	 2.03<br>8.02<br>0.35
Ramachandran plot<br>Favored (%)<br>Allowed (%)<br>Disallowed (%)	 88.43<br>11.13<br>0.44

CC (mask)	0.86
CC (box)	0.89
CC (peaks)	0.75
CC (volume)	0.85
